# IFN-β Acts on Monocytes to Ameliorate CNS Autoimmunity by Inhibiting Proinflammatory Cross-Talk Between Monocytes and Th Cells

**DOI:** 10.3389/fimmu.2021.679498

**Published:** 2021-06-04

**Authors:** Javad Rasouli, Giacomo Casella, Larissa L. W. Ishikawa, Rodolfo Thome, Alexandra Boehm, Adam Ertel, Carolina R. Melo-Silva, Elisabeth R. Mari, Patrizia Porazzi, Weifeng Zhang, Dan Xiao, Luis J. Sigal, Paolo Fortina, Guang-Xian Zhang, Abdolmohamad Rostami, Bogoljub Ciric

**Affiliations:** ^1^Department of Neurology, Thomas Jefferson University, Philadelphia, PA, United States; ^2^Sidney Kimmel Cancer Center, Department of Cancer Biology, Thomas Jefferson University, Philadelphia, PA, United States; ^3^Department of Microbiology and Immunology, Thomas Jefferson University, Philadelphia, PA, United States; ^4^Department of Translation and Precision Medicine, Sapienza University, Rome, Italy

**Keywords:** multiple sclerosis, EAE, GM-CSF, IFN-β, Th cells, monocytes

## Abstract

IFN-β has been the treatment for multiple sclerosis (MS) for almost three decades, but understanding the mechanisms underlying its beneficial effects remains incomplete. We have shown that MS patients have increased numbers of GM-CSF^+^ Th cells in circulation, and that IFN-β therapy reduces their numbers. GM-CSF expression by myelin-specific Th cells is essential for the development of experimental autoimmune encephalomyelitis (EAE), an animal model of MS. These findings suggested that IFN-β therapy may function *via* suppression of GM-CSF production by Th cells. In the current study, we elucidated a feedback loop between monocytes and Th cells that amplifies autoimmune neuroinflammation, and found that IFN-β therapy ameliorates central nervous system (CNS) autoimmunity by inhibiting this proinflammatory loop. IFN-β suppressed GM-CSF production in Th cells indirectly by acting on monocytes, and IFN-β signaling in monocytes was required for EAE suppression. IFN-β increased IL-10 expression by monocytes, and IL-10 was required for the suppressive effects of IFN-β. IFN-β treatment suppressed IL-1β expression by monocytes in the CNS of mice with EAE. GM-CSF from Th cells induced IL-1β production by monocytes, and, in a positive feedback loop, IL-1β augmented GM-CSF production by Th cells. In addition to GM-CSF, TNF and FASL expression by Th cells was also necessary for IL-1β production by monocyte. IFN-β inhibited GM-CSF, TNF, and FASL expression by Th cells to suppress IL-1β secretion by monocytes. Overall, our study describes a positive feedback loop involving several Th cell- and monocyte-derived molecules, and IFN-β actions on monocytes disrupting this proinflammatory loop.

## Introduction

Multiple sclerosis (MS) is an immune-mediated demyelinating disease characterized by the accumulation of immune cells in the central nervous system (CNS) (1). While the etiology of MS remains elusive, our view on the pathophysiology of MS has largely evolved through analogy with experimental autoimmune encephalomyelitis (EAE), an animal model of MS. It is widely accepted that in MS and EAE, myelin-specific helper T (Th) cells primed in the peripheral lymphoid organs enter the CNS and initiate inflammation through interaction with antigen-presenting cells (APCs). APCs present myelin antigens (Ags) and reactivate CNS-infiltrated myelin-specific Th cells (2–5). Reactivated Th cells start producing pro-inflammatory mediators, such as granulocyte macrophage-colony stimulating factor (GM-CSF), which license the inflammatory phenotype of monocytes. Monocytes aggravate the CNS inflammation by producing pro-inflammatory cytokines, chemokines, reactive oxygen species, and reactive nitrogen species that lead to myelin damage and neuronal loss (6, 7).

IFN-β signals through type-I IFN receptor (IFNAR1) and activates the JAK/STAT pathway that mediates intracellular effects of IFN-β (8). Mice lacking IFNAR1 develop more severe EAE compared to wild-type (WT) controls (9), and treatment with recombinant IFN-β (rIFN-β) ameliorates EAE (10). In 1993, injectable rIFN-β became the first FDA-approved disease-modifying therapy for MS (11, 12). Despite extensive study, the mechanisms whereby rIFN-β ameliorates MS remain incompletely understood; however, it is known that rIFN-β downregulates expression of HLA Class II, thereby inhibiting Th cell activation (13), and that it reduces expression of pro-inflammatory cytokines and molecules that facilitate extravasation of immune cells, such as matrix metalloproteases and very late antigen-4 (VLA-4) (14). Interestingly, it has been shown that rIFN-β ameliorates EAE driven by Th1 cells, but, in contrast, it exacerbates disease induced by Th17 cells (15). The therapeutic effect in Th1-induced EAE correlated with increased IL-10 production, whereas in Th17-induced disease, IL-10 production was unaltered by rIFN-β treatment, suggesting that an increase in IL-10 production is required for EAE suppression by rIFN-β (10).

GM-CSF is a pro-inflammatory cytokine essential to the development and progression of EAE (6, 16–18). It is thought that GM-CSF plays a similarly important role in MS, and positive outcomes of a clinical trial that targeted GM-CSF in MS support this view (19). Receptor for GM-CSF is expressed by both myeloid cells and tissue resident cells, but not by lymphoid cells, such as T cells (20–25). Although different subsets of myeloid cells express the receptor, only expression by monocytes is necessary for EAE to develop (6). Th cells are the relevant source of GM-CSF in EAE, and their encephalitogenicity depends on the capacity to produce GM-CSF (26–28). Recent studies have shown increased frequencies of GM-CSF^+^ Th cells in the periphery and cerebrospinal fluid (CSF) of MS patients compared to healthy individuals, and treatments with some immunomodulatory therapies, such as rIFN-β, normalize their numbers (29–32). Overall, GM-CSF has been identified as a key mediator of autoimmune neuroinflammation and approaches that target its production or bioactivity are considered a viable strategy for therapy of MS.

Given the crucial role of T cell-produced GM-CSF in EAE, and observations that rIFN-β therapy reduces its production, we elucidated the mechanisms underlying this effect. rIFN-β treatment suppressed GM-CSF expression and EAE development by acting on monocytes, which adopted anti-inflammatory phenotype. rIFN-β induced IL-10 and TGF-β expression in monocytes, and these cytokines were required for suppression of GM-CSF production by Th cells. rIFN-β also suppressed IL-1β expression by monocytes by reducing GM-CSF, TNF, and FASL expression by Th cells. Our study reveals that rIFN-β action on monocytes disrupts a positive feedback loop between monocytes and Th cells that otherwise amplifies production of proinflammatory mediators GM-CSF and IL-1β.

## Materials and Methods

### Mice and EAE Induction

C57BL/6, CD45.1, *Ifnar1*^-/-^, *Stat2*^-/-^, *Il10*^-/-^, *Il10rb*^-/-^, *Il1r1*^-/-^, *Caspase-1*^-/-^, *Ccr2*^-/-^, *Csf2*^-/-^, *Il27ra*^-/-^, GFP^+^, and *Rag1*^-/-^ mice were purchased from Jackson Laboratory (Bar Harbor, ME, USA). *Stat1*^-/-^ mice were purchased from Taconic Farms (Hudson, NY, USA). To generate *LysM-Cre^+^Ifnar1*^fl/fl^ mice, *LysM-Cre* mice were crossed with *Ifnar1*^fl/fl^ mice and used for EAE experiments. Mice were kept in specific pathogen–free conditions in 12/12 h of light/dark cycles with food and water *ad libitum*. All experimental breeding and procedures were performed with the approval of the Institutional Animal Care and Use Committee of Thomas Jefferson University.

Mice were immunized for EAE induction by subcutaneous injection of 200 µg MOG_35-55_ (Genscript, CA, USA) emulsified in CFA. Mice received 200 ng of pertussis toxin (Sigma-Aldrich, MO, USA) on days 0 and 2 p.i. and were scored and weighed daily. Mice were scored according to the following scale: 0, no sign of clinical disease; 1, paresis of the tail; 2, paresis of one hind limb; 3, paresis of both hind limbs; 4, paresis of the abdomen; 5, moribund/death.

### Cell Culture

Naïve (CD62L^+^CD44^-^CD25^-^CD4^+^) or effector/memory (CD62L^-^CD44^hi^CD25^-^CD4^+^) T cells were FACS sorted and cultured at a ratio of 1:4 with T cell-depleted splenocytes, CD11b^+^, CD11c^+^, or CD19^+^ cells at a density of 1x10^6^ cell/ml. Cells were activated with anti-CD3/28 (1 µg/ml; BioXCell) in the presence or absence of rIFN-β (1000 IU/ml; R&D Systems). Cells were also treated with IL-1β (20 ng/ml; PeproTech), GM-CSF (20 ng/ml; R&D Systems), TNF (20 ng/ml; R&D Systems), anti-IL-1R1 (10 µg/ml; BioXCell), anti-TNF (10 µg/ml; BioXCell), anti-GM-CSF (10 µg/ml; Biolegend), anti-FASL (10 µg/ml; BioXCell) antibodies, to study their effect on cytokine expression by T cells or APCs.

Splenocytes from PBS- or IFN-β-treated mice with EAE at day 8 p.i. were cultured with MOG_35-55_ (25 µg/ml), at a density of 2 x 10^6^ cells/ml. Cell culture supernatants were collected for cytokine quantification by ELISA.

To study the effect of caspase-8 activation in IL-1β production by myeloid cells, WT naive CD4^+^ T cells were cultured with CD11b^+^ cells at a density of 1 x 10^6^ cells/ml. Cells were activated with anti-CD3/28 (1 µg/ml) with DMSO or caspase-8 inhibitor (20 µM; R&D Systems).

Bone marrow (BM) cells from WT and *Il27ra*^-/-^ mice were differentiated into macrophages for 6 days with M-CSF (100 ng/ml, R&D Systems) as described previously (33). Cells were plated at 5 x 10^5^ in MW6 petri dishes (Becton Dickinson, San Diego, CA, USA) and were treated with PBS, or rIFN-β (1000 IU/ml) for 24 h. Cell culture supernatants were harvested for cytokine quantification by ELISA and cells were processed for RNA extraction.

### Isolation of CNS Mononuclear Cells

CNS mononuclear cells were isolated as previously described (34). In brief, mice with EAE were perfused with ice-cold PBS, brains and spinal cords were collected, minced, and digested in Liberase (Sigma-Aldrich) for 30 min at 37°C. Digested CNS tissues were further mechanically dissociated, and mononuclear cells were isolated using Percoll gradient (GE Healthcare).

### Flow Cytometry and Intracellular Staining

Cells either from mice with EAE or *in vitro* culture were activated with 50 ng/ml Phorbol 12-myristate 13-acetate (PMA) (Sigma-Aldrich), 500 ng/ml ionomycin (Sigma-Aldrich), and 1µg/ml of GolgiPlug (BD Biosciences) for four hours. Cells were washed and stained with surface antibodies (**Supplementary Table 1**) for 20 min at 4°C. Cells were washed, fixed and permeabilized with Caltag Fix/Perm reagents (Invitrogen) following the manufacturer’s instructions and stained with intracellular antibodies as listed in **Supplementary Table 1**. For IL-1β (pro-IL-1β) staining, CNS mononuclear cells were activated with PMA/Ionomycin/GolgiPlug for 6 h and stained with surface and intracellular antibodies. Data were acquired on a FACSAria Fusion (BD Biosciences) and analyzed using FlowJo software (TreeStar).

### Cytokine Quantification

Cell culture supernatants were collected, and GM‐CSF, IFN-γ, IL-17A, IL-10, IL-1β, IL-1α, and TNF concentrations were measured by ELISA (R&D Systems) according to the manufacturer’s instructions.

### RT-PCR

RNA was extracted from cells of mice with EAE or *in vitro* cell culture using RNeasy Plus Mini Kit (Qiagen). cDNA was then converted and PCR was performed using the following FAM conjugated primer‐probe mixtures (Applied Biosystems): *Csf2* (Mm01290062_m1), *Ifng*, (Mm01168134_m1), *Il6* (Mm00446190_m1), *Il1a* (Mm00439620_m1), *Il1b* (Mm0001336189_m1), *Il12a* (Mm00434169_m1), *Il23a* (Mm00518984_m1), *Il27* (Mm00461162_m1), *Tgfb1* (Mm011178820_m1), *Il10* (Mm00439614_m1), *Ifnb1* (Mm00439552_m1), *Irf7* (Mm00516788_m1), *Tlr9* (Mm00446193_m1), and *Siglec1* (Mm00488332_m1). Values were normalized to VIC conjugated *Gapdh* (Mm99999915_g1) and compared to control samples.

### EAE in *Rag1^-/-^* Mice

In order to study the role of IL-1R1 in GM-CSF expression by CD4^+^ T cells, total CD4^+^ T cells from WT (CD45.1) and *Il1r1^-/-^* (CD45.2) mice were purified using a CD4 isolation kit (Miltenyi Biotec). CD4^+^ T cells from WT and *Il1r1^-/-^* mice were mixed at a 1:1 ratio and 1 x 10^7^ cells were transferred to *Rag1*^-/-^ recipient mice. Three days past transfer, *Rag1*^-/-^ mice were immunized for EAE induction.

### Bone Marrow Chimerism

WT recipient mice were lethally irradiated with 2 x 550 Rad with a 12 h interval between irradiations. Mice received 5 x 10^6^ BM cells from WT (CD45.1) and *Ccr2*^-/-^ (CD45.2) mice in a 1:1 ratio (total 1 x 10^7^ cells), or from *Ifnar1*^-/-^ and *Ccr2*^-/-^ mice. For RNA-seq analysis experiment, irradiated GFP^+^ mice received a 1:1 mixture of BM cells from WT (CD45.1) and *Ifnar1*^-/-^ (CD45.2) mice. Chimera mice were used for EAE induction 6-7 weeks after BM cell transfer.

### RNA-seq Analysis

WT/*Ifnar1*^-/-^ chimeric mice were immunized for EAE induction and treated with rIFN-β daily. At disease peak, WT (CD45.1) and *Ifnar1*^-/-^ (CD45.2) monocytes (GFP^-^CD45^+^CD11b^+^Ly6C^hi^Ly6G^-^) were FACS sorted from the CNS of mice, and RNA was extracted using RNeasy Plus Mini kit (Qiagen), according to the manufacturer’s instructions.

100 ng of total RNA was used to prepare libraries using TruSeq Stranded Total RNA kit (Illumina) following the manufacturer’s protocol. The final libraries at a concentration of 4 nM were sequenced on NextSeq 500 using 75bp paired-end chemistry. Raw FASTQ sequencing reads were mapped against the reference genome of Mus musculus Ensembl version GRCm38 utilizing further information from the gene transfer format (.gtf) annotation from GENCODE version GRCh38.p12 (for mouse) using RSEM. Total read counts, and normalized Transcripts Per Million (TPM) were obtained using RSEM’s calculate-expression function. Beforehand, differential expression, batch effects or sample heterogeneity were tested using iSeqQC (https://github.com/gkumar09/iSeqQC). Differential gene expression was tested using the DESeq2 package in R/Bioconductor. Genes were considered differentially expressed (DE) if they had adjusted p ≤ 0.05 and absolute fold change ≥ 1.5. All the plots were constructed using R/Bioconductor.

Gene Set Enrichment Analysis (GSEA) was performed on DESeq2 normalized count data using the default signal-to-noise metric (35). The MSigDB mouse ENSEMBL to human ortholog CHIP file (Mouse_ENSEMBL_Gene_ID_Human_ Orthologs_MSigDB.v7.2) was used for mouse to human gene ID conversion (36). The gene sets analyzed include the MSigDB C7: immunologic signature gene sets, as well as custom monocyte and plasmacytoid dendritic cell gene sets derived from Villani 2017 and Rodrigues 2018 (37, 38). Plots were generated using GSEA normalized enrichment score (NES), and false discovery rate (FDR) q-value.

### Statistical Analysis

Statistical analysis was performed by GraphPad Prism 8 software. EAE clinical scores were analyzed using Two-way ANOVA, and Bonferroni correction test was used to adjust *p* values for multiple comparisons. The paired, two-tailed Student *t*-test was used to analyze transferred WT and *Il1r1*^-/-^ CD4^+^ T cells within the same recipient mouse. Parametric data were analyzed using an unpaired, two-tailed Student t-test. In some experiments, one-way ANOVA with Bonferroni correction was applied for adjustment of the significance values for multiple comparisons; adjusted p ≤ 0.05 was considered significant. Data shown are mean ± SEM.

## Results

### rIFN-β Treatment Decreases the Numbers of GM-CSF-Producing Th Cells in the Periphery and CNS of Mice with EAE

**Figure 1:**
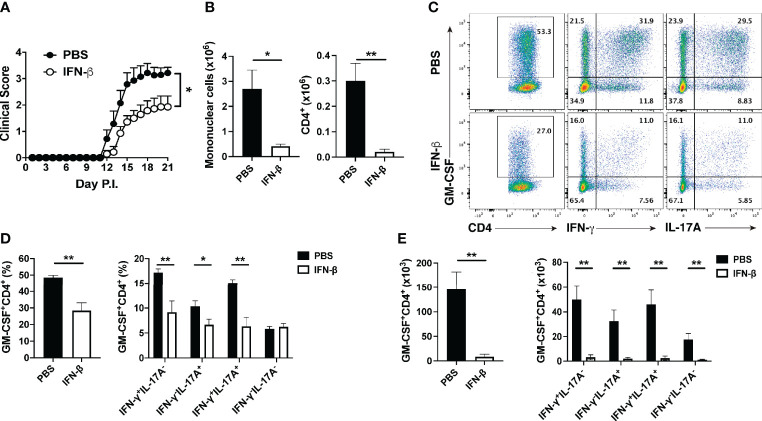
rIFN-β decreases the numbers of GM-CSF-producing Th cells in the CNS of mice with EAE. Female C57BL/6 mice (n=7 per group) were immunized for EAE induction with MOG_35-55_ and received daily intraperitoneal (i.p.) injections of PBS or rIFN-β (30000 IU). Mice were scored daily for clinical signs and sacrificed at disease peak (day 15 p.i.), and CNS mononuclear cells were analyzed by flow cytometry. **(A)** Clinical severity score. **(B)** Total numbers of CNS mononuclear cells and CD4^+^ T cells in the CNS at day 15 p.i. **(C)** Representative flow cytometry dot plots showing GM-CSF, IFN-γ, and IL-17A expression by CD4^+^ T cells in the CNS at day 15 p.i. **(D)** Proportions and **(E)** total numbers of GM-CSF^+^CD4^+^, and different subsets of GM-CSF^+^ CD4^+^ T cells in the CNS. These experiments were conducted twice with similar outcomes. Data shown are mean ± SEM. For EAE, p-values were calculated using two-way ANOVA with Bonferroni’s multiple comparison correction. Parametric datasets were analyzed using unpaired Student’s *t*-test; *p < 0.05, **p < 0.01.

We and others have shown that MS patients have greater numbers of GM-CSF^+^ Th cells in peripheral blood than healthy subjects, and that rIFN-β therapy reduces their numbers (29–31). We therefore hypothesized that the beneficial effects of rIFN-β in EAE and MS are mediated by inhibition of GM-CSF production by Th cells. To test this hypothesis, we immunized mice for EAE induction and treated them daily with rIFN-β. Consistent with previous reports (10), the treatment reduced severity of clinical disease without substantially delaying its onset (**Figure 1A**). Mice were sacrificed at days 8 and 15 post immunization (p.i.), and their immune cells from the spleen and CNS were profiled by flow cytometry. Analysis of splenocytes on day 8 p.i. showed that rIFN-β treatment reduced both frequencies of GM-CSF^+^ cells among CD4^+^ T cells and their total numbers in splenocytes, with the reduction in their total numbers occurring across multiple subsets of Th cells (**Supplementary Figure 1A**). We next evaluated ex vivo the effect of rIFN-β treatment on cytokine production by splenocytes of rIFN-β-treated mice in comparison to control mice (from day 8 p.i.). Splenocytes from rIFN-β-treated mice activated with myelin Ag secreted less GM-CSF and IL-17A in culture supernatants, whereas reduction in IFN-γ secretion did not reach statistical significance (**Supplementary Figure 1B**). We also determined the effect of rIFN-β treatment on the CNS-infiltrated T cells at disease peak (day 15 p.i.). rIFN-β treatment reduced total numbers of mononuclear cells, including CD4^+^ T cells (**Figure 1B**). rIFN-β-treated mice had reduced numbers of total GM-CSF^+^CD4^+^ T cells and reduced frequencies of GM-CSF^+^ cells in CD4^+^ T cells, including in: Th1 (IFN-γ^+^IL-17A^-^), Th17 (IFN-γ^-^IL-17A^+^), Th1/17 (IFN-γ^+^IL-17A^+^), and ThGM (IFN-γ^-^IL-17A^-^) (28) cells (**Figures 1C–E**). These results show that rIFN-β treatment reduces both frequencies and numbers of GM-CSF^+^CD4^+^ T cells in the periphery and CNS of mice with EAE.

### rIFN-β Suppresses GM-CSF Production by Th Cells *In Vitro* by Acting on APCs

**Figure 2:**
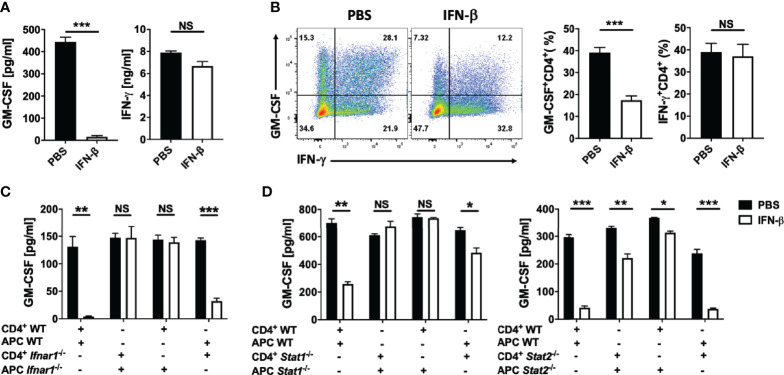
rIFN-β acts on antigen presenting cells to suppress GM-CSF production by Th cells. Splenic naïve (CD62^+^CD44^-^CD25^-^) CD4^+^ T cells were co-cultured with T cell-depleted splenocytes (APCs) and activated for 72 h with anti-CD3/28 Abs, with or without addition of rIFN-β (1000 IU/ml) into culturing media. **(A)** GM-CSF and IFN-γ concentrations in cell culture supernatants measured by ELISA after three days of culturing. **(B)** Representative flow cytometry dot plots showing GM-CSF and IFN-γ expression by CD4^+^ T cells, and proportions (%) of GM-CSF^+^ and IFN-γ^+^ CD4^+^ T cells. **(C)** WT or *Ifnar1*^-/-^ splenic naïve CD4^+^ T cells were co-cultured with WT or *Ifnar1*^-/-^ APCs and activated for 72 h with anti-CD3/28 Abs, with or without addition of rIFN-β into culturing media. GM-CSF concentrations in cell culture supernatants were measured by ELISA after three days of culturing. **(D)** WT, *Stat1*^-/-^ or *Stat2*^-/-^ splenic naïve CD4^+^ T cells and APCs were co-cultured, activated with anti-CD3/28 Abs, with or without addition of rIFN-β into culturing media. GM-CSF concentrations in cell culture supernatants were measured by ELISA after 72 h of culturing. These experiments were conducted three times with similar outcomes. Data shown are mean ± SEM. P-values were calculated using unpaired Student’s *t*-test; *p < 0.05, **p < 0.01, ***p < 0.001, NS, not significant.

To begin studying the mechanisms whereby rIFN-β suppresses GM-CSF expression by Th cells, we first tested its effect in co-cultures of naïve CD4^+^ T cells and APCs. rIFN-β suppressed GM-CSF production in a dose-dependent manner (**Supplementary Figure 2A**). GM-CSF production by naïve CD4^+^ T cells was upregulated 48 h after activation (**Supplementary Figure 2B**), and was strongly suppressed by rIFN-β, without an effect on IFN-γ production (**Figure 2A**). rIFN-β significantly decreased the proportions of GM-CSF^+^ Th cells, while the frequencies of IFN-γ^+^ Th cells remained unchanged (**Figure 2B**). Given that rIFN-β suppressed GM-CSF production by recently activated naive CD4^+^ T cells, we next tested whether it has a similar effect on Ag-experienced effector/memory CD4^+^ T (T_EM_) cells. Indeed, rIFN-β suppressed GM-CSF production by T_EM_ cells similar to naïve CD4^+^ T cells (**Supplementary Figure 2C**). These results show that rIFN-β strongly suppresses GM-CSF expression by all CD4^+^ T cells *in vitro*, suggesting that rIFN-β affects Th cell functions in both priming and effector phases of an immune response.

To determine whether rIFN-β suppresses GM-CSF production by Th cells by directly acting on them, we co-cultured WT or *Ifnar1*^-/-^ naïve CD4^+^ T cells and APCs. rIFN-β suppressed GM-CSF expression only in samples with WT APCs, whereas *Ifnar1* expression by CD4^+^ T cells was inconsequential (**Supplementary Figure 2D** and **Figure 2C**). IFN-β signaling through IFNAR1 leads to phosphorylation of STAT1 and STAT2, which then mediate intracellular effects of IFN-β (8). We confirmed that the effect of rIFN-β on GM-CSF production by Th cells is indirect by co-culturing WT, *Stat1*^-/-^ or *Stat2*^-/-^ naïve CD4^+^ T cells and APCs. Similar to the result from *Ifnar1*^-/-^/WT cell co-cultures, rIFN-β suppressed GM-CSF production only in samples with WT APCs, whereas the lack of STAT1 or STAT2 (**Figure 2D**) in Th cells was inconsequential. We confirmed that the effect of rIFN-β on GM-CSF-producing Th cells is indirect by treating isolated total CD4^+^ T cells with rIFN-β. rIFN-β failed to suppress GM-CSF production by Th cells in the absence of APCs (**Supplementary Figure 2E**), demonstrating that rIFN-β suppresses GM-CSF production by Th cells indirectly, by acting on APCs.

### rIFN-β Acts on Myeloid Cells to Suppress EAE

**Figure 3:**
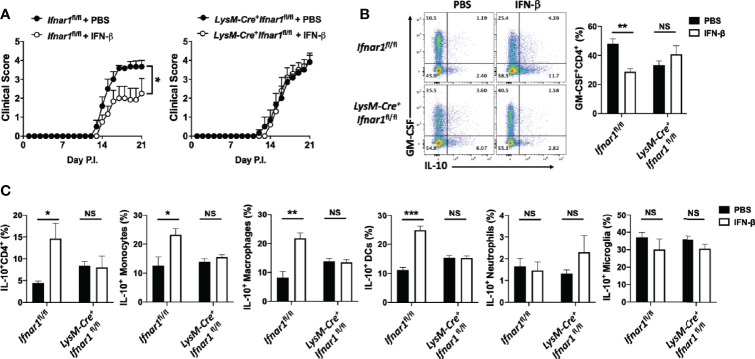
rIFN-β acts on myeloid cells to suppress the development of EAE. *Ifnar1*^fl/fl^ and *LysM-Cre^+^Ifnar1*^fl/fl^ mice (n=6 per group) were immunized for EAE induction with MOG_35-55_ and received daily i.p. injections of PBS or rIFN-β, sacrificed at day 21 p.i., and CNS cells were analyzed by flow cytometry. **(A)** Clinical severity score. **(B)** Representative flow cytometry dot plots showing GM-CSF and IL-10 expression by CD4^+^ T cells, and proportions (%) of GM-CSF^+^CD4^+^ T cells from the CNS. **(C)** Proportions (%) of IL-10^+^ CD4^+^ T cells, monocytes (CD45^hi^CD11b^+^Ly6C^hi^Ly6G^-^), DCs (CD45^hi^CD11b^-^CD11c^+^MHCII^+^), macrophages (CD45^hi^CD11b^+^F4/80^+^Ly6C^-^Ly6G^-^), neutrophils (CD45^hi^CD11b^+^Ly6C^low^Ly6G^+^), and microglia (CD45^int^CD11b^+^) in the CNS. EAE experiments were conducted at least two times with similar outcomes. Data shown are mean ± SEM. For EAE, p-values were calculated using two-way ANOVA with Bonferroni’s multiple comparison correction. Parametric datasets were analyzed using unpaired Student’s *t*-test; *p < 0.05, **p < 0.01, ***p < 0.001, NS, not significant.

rIFN-β suppressed GM-CSF production by Th cells *in vitro via* its action on APCs. To determine whether rIFN-β suppresses EAE by acting on APCs, we generated mice (*LysM-Cre^+^Ifnar1*^fl/fl^) in which most myeloid cells lack IFNAR1. Cre expression driven by M lysosome (LysM) results in loss of IFNAR1 expression by monocytes, macrophages, and granulocytes, but does not affect its expression by pDCs and conventional DCs (9). We immunized *LysM-Cre^+^Ifnar1*^fl/fl^ mice for EAE induction and treated them with rIFN-β daily. The treatment suppressed EAE in control mice (*Ifnar1*^fl/fl^), whereas it had no effect in *LysM-Cre^+^Ifnar1*^fl/fl^ mice (**Figure 3A**). Consistent with this, rIFN-β decreased the frequencies of GM-CSF^+^ Th cells in the CNS of control mice, but it failed to do so in *LysM-Cre^+^Ifnar1*^fl/fl^ mice (**Figure 3B**). In addition, rIFN-β increased the frequencies of IL-10^+^ Th cells in the CNS of control mice, but not in *LysM-Cre^+^Ifnar1*^fl/fl^ mice (**Figures 3B, C**). We also analyzed IL-10 expression by different subsets of APCs in the CNS. rIFN-β treatment in control mice increased the frequencies of IL-10^+^ monocytes, DCs, and macrophages, but not of IL-10^+^ neutrophils and microglia. In contrast, rIFN-β did not alter the frequencies of IL-10^+^ APCs in *LysM-Cre^+^Ifnar1*^fl/fl^ mice (**Figure 3C**). Treatment of BM-derived macrophages (BMDMs) confirmed that rIFN-β can directly induce IL-10 expression by myeloid cells (**Supplementary Figure 2F**). These results show that rIFN-β suppresses EAE by acting on myeloid cells.

### rIFN-β Suppresses EAE Development by Acting on Monocytes

**Figure 4:**
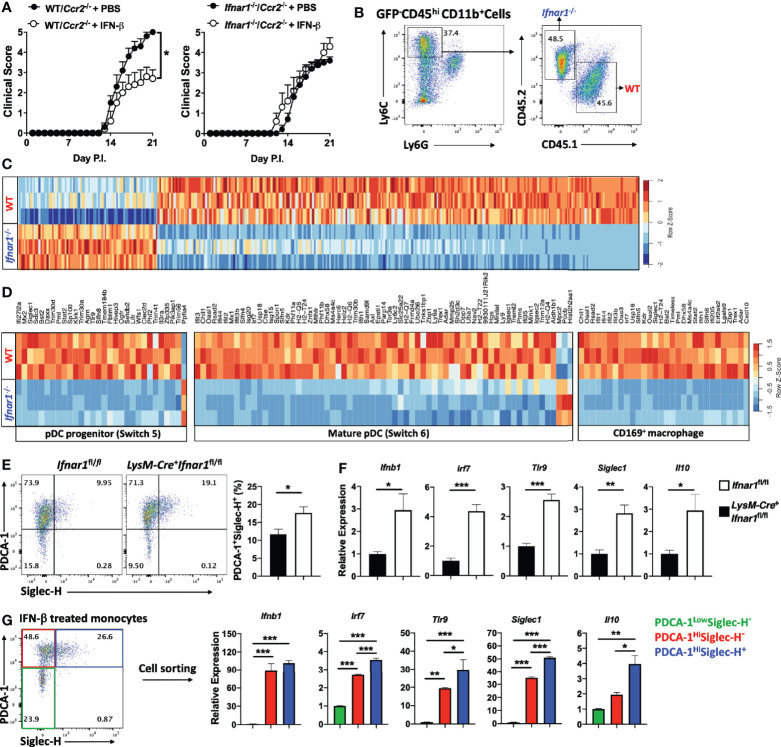
rIFN-β suppresses EAE development by acting on monocytes. **(A)** To generate mixed BM chimera mice (n=5 per group), BM from WT (CD45.1) or *Ifnar1*^-/-^ mice were mixed with BM from *Ccr2*^-/-^ mice in a 1:1 ratio (total 1 x 10^7^ cells) and injected into lethally irradiated WT (CD45.2) recipient mice. After seven weeks, mice were immunized for EAE induction and treated daily with i.p. injections of PBS or rIFN-β. Clinical severity scores of WT/*Ccr2*^-/-^ and *Ifnar1*^-/-^/*Ccr2*^-/-^ chimera mice are shown. **(B)** Mixed BM chimera mice were generated by transferring BM from WT (CD45.1) and *Ifnar1*^-/-^ (CD45.2) mice in a 1:1 ratio (total 1 x 10^7^ cells) into lethally irradiated GFP^+^ recipient mice (n=3). Seven weeks later, mice were immunized for EAE induction and treated with rIFN-β daily. Mice were sacrificed at day 15 p.i. and monocytes (CD45^hi^CD11b^+^Ly6C^hi^Ly6G^-^) were FACS sorted from CNS mononuclear cells. Representative flow cytometry dot plots showing FACS sorting strategies. **(C)** RNA from WT and *Ifnar1*^-/-^ monocytes (n=3 per group) was extracted and analyzed by RNA-seq. Hierarchical clustering for WT and *Ifnar1*^-/-^ monocyte-specific genes. **(D)** GSEA enrichment analysis of WT and *Ifnar1*^-/-^ monocyte-specific genes in comparison with GSE114315 and SRA#SUB2908162. Heatmap showing pDC progenitor-, mature pDC- and CD169^+^ macrophage-specific genes in rIFN-β-treated WT and *Ifnar1*^-/-^ monocytes. **(E)** *Ifnar1*^fl/fl^ and LysM-Cre^+^*Ifnar1*^fl/fl^ mice (n=5 per group) were immunized for EAE induction and treated with rIFN-β daily. Mice were sacrificed at day 21 p.i. and CNS-infiltrated monocytes were FACS sorted for flow cytometry and gene expression analysis. Representative flow cytometry dot plots showing PDCA-1 and Siglec-H expression by monocytes. Proportions (%) of PDCA-1^+^ Siglec-H^+^ monocytes. **(F)** RNA from *Ifnar1*^fl/fl^ and LysM-Cre^+^*Ifnar1*^fl/fl^ monocytes was extracted and gene expression determined using RT-PCR. **(G)** Mice (n=3) were immunized for EAE induction and sacrificed at day 21 p.i. CNS mononuclear cells were isolated and cultured with MOG_35-55_ and rIFN-β (1000 IU/ml) for 24 h. Monocytes were FACS sorted based on PDCA-1 and Siglec-H expression and RNA was extracted for gene expression analysis by RT-PCR. Data shown are mean ± SEM. For EAE mice, p-values were calculated using two-way ANOVA with Bonferroni’s multiple comparison correction. P-values were calculated using unpaired Student’s *t*-test in **(E, F)**, and one-way ANOVA with Bonferroni’s multiple comparisons test in **(G)**; ***p < 0.05, **p < 0.01, ***p < 0.001.

Given the prominent role of monocytes in the pathophysiology of EAE (39), we hypothesized that among myeloid cells, rIFN-β action on monocytes leads to EAE suppression. To test this possibility, we generated mixed BM chimeras in which recipient mice received half BM cells from *Ifnar1*^-/-^ mice and half from *Ccr2*^-/-^ mice. *Ccr2*^-/-^ monocytes fail to leave BM (40); therefore, virtually all monocytes in the circulation of mixed BM chimera mice were of *Ccr2^+/+^* (*Ifnar1*^-/-^) origin. Control mixed BM chimera mice were generated with BM from WT mice (CD45.1) and *Ccr2*^-/-^ mice (CD45.2). After 7 weeks of reconstitution, we confirmed that almost all monocytes in the periphery of chimera mice were of WT origin (**Supplementary Figures 3A, B**). Chimera mice were then immunized for EAE induction and received a daily dosage of rIFN-β for three weeks (**Supplementary Figure 3A**). rIFN-β treatment suppressed disease development in mice with WT BM, whereas it failed to suppress disease in mice with *Ifnar1*^-/-^ BM (**Figure 4A**). These results demonstrate that IFNAR1 signaling in monocytes leads to disease suppression by rIFN-β.

### rIFN-β Treatment Induces IFN-β Expression in CNS-Infiltrated Monocytes

To study the direct effect of rIFN-β on monocytes during EAE, we used a mixed BM chimera model in which irradiated recipient mice (GFP mice) received half BM cells from WT (CD45.1) and half from *Ifnar1*^-/-^ (CD45.2) mice. Chimera mice were 7 weeks later immunized for EAE induction and treated with rIFN-β (**Supplementary Figure 4A**). Mice were sacrificed at disease peak, GFP^-^ WT and *Ifnar1*^-/-^ inflammatory monocytes (CD45^hi^CD11b^+^Ly6C^hi^Ly6G^-^) from the CNS were FACS sorted (**Supplementary Figure 4B**), and their transcriptomes characterized by RNA-seq (**Figure 4B**). 381 genes were differentially expressed in WT monocytes, with 292 genes being upregulated and 89 genes downregulated (**Supplementary Table 2**). Among upregulated genes were several [*Irf7*, *Tlr9*, and *Bst2* (PDCA-1)] (**Figure 4C**) known to induce type-I IFN expression in some immune cells, including pDCs (41). We therefore compared our transcriptomes of monocytes with the list of pDC-specific genes extracted from accession no. GSE114315 (38). Gene set enrichment analysis (GSEA) showed that rIFN-β induced in monocytes a transcriptional profile enriched for both pDC progenitor and mature pDCs genes (**Supplementary Figure 4C** and **Figure 4D**). In addition, rIFN-β induced *Siglec1* (CD169) (**Figure 4D**), which is associated with macrophages that produce type-I IFNs during an infection (42). We therefore compared transcriptomes of WT and *Ifnar1*^-/-^ monocytes with the set of CD169^+^ macrophage genes extracted from SRA#SUB2908162 (42). GSEA revealed that rIFN-β in WT monocytes induced a transcriptional profile enriched for genes characteristic for CD169^+^ macrophages (**Supplementary Figure 4D** and **Figure 4D**).

To further test whether rIFN-β induces the pDC-like phenotype in monocytes, we immunized *Ifnar1*^fl/fl^ and *LysM-Cre^+^Ifnar1*^fl/fl^ mice for EAE induction, treated them with rIFN-β, and analyzed monocytes from the CNS for pDC markers. rIFN-β treatment increased the frequencies of PDCA-1^+^SiglecH^+^ monocytes from *Ifnar1*^fl/fl^ mice compared to those from *LysM-Cre^+^Ifnar1*^fl/fl^ mice (**Figure 4E**), and upregulated their expression of *Ifnb1*, *Irf7*, *Tlr9*, *siglec1*, and *Il10* (**Figure 4F**). We also activated with MOG_35-55_ mononuclear cells from the CNS of WT mice with EAE in the presence of rIFN-β for 24 h. Similar to results from rIFN-β-treated mice, *in vitro*-treated monocytes had higher frequencies of PDCA-1^+^Siglec-H^+^ cells (**Supplementary Figure 4E**), and higher expression of *Ifnb1*, *Irf7*, *Tlr9*, *siglec1*, and *Il10* than control monocytes (**Supplementary Figure 4F**). In addition to increasing the frequencies of PDCA-1^+^Siglec-H^+^ monocytes, rIFN-β upregulated levels of both PDCA-1 and Siglec-H per cell *in vivo* and *in vitro* (**Supplementary Figures 4G, H**). We also tested the effect of rIFN-β treatment *in vivo* and *in vitro* on IFN-α expression by monocytes, but non-reproducible outcomes among repeats precluded drawing any conclusions (data not shown). Overall, these results demonstrate that rIFN-β treatment induces type I-IFNs-expressing phenotype in monocytes.

To identify a subset of monocytes that expresses type-I IFNs, we treated monocytes from the CNS of mice with EAE with rIFN-β and sorted three populations based on PDCA-1 and Siglec-H expression. PDCA-1^Hi^ monocytes, irrespective of Siglec-H expression, had elevated expression of *Ifnb1*, *Irf7*, *Tlr9*, and *Siglec1* compared with PDCA-1^Low^ monocytes, whereas only PDCA-1^Hi^Siglec-H^+^ monocytes upregulated *Il10* expression (**Figure 4G**). These results show that PDCA-1 expression identifies monocytes that produce IFN-β, and that simultaneous expression of PDCA-1 and Siglec-H correlates with co-expression of IFN-β and IL-10.

### rIFN-β Ameliorates EAE *via* IL-10, but Independent of IL-27

To identify the mechanisms whereby rIFN-β modulates APCs to suppress GM-CSF production by Th cells, we co-cultured naïve CD4^+^ T cells and APCs with addition of rIFN-β, and quantified the expression of cytokines known to regulate GM-CSF expression. rIFN-β strongly suppressed expression of IL-1α and IL-1β, and to a lesser extent of IL-12, IL-23, IL-6 and IFN-γ, but it upregulated IL-10 expression, which was readily detectable 48 h after activation, peaking at 72 h (**Supplementary Figures 5A, B**).

**Figure 5:**
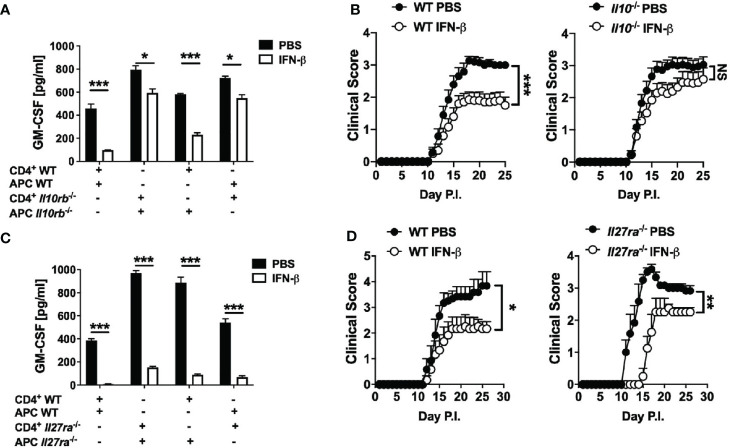
rIFN-β ameliorates EAE *via* IL-10, but independently of IL-27. Splenic WT or *Il10rb*^-/-^ naïve CD4^+^ T cells were co-cultured with of WT or *Il10rb*^-/-^ APCs and activated with anti-CD3/28 Abs with or without addition of rIFN-β (1000 IU/ml) into culturing media for 72 h. **(A)** GM-CSF concentrations in cell culture supernatants were measured by ELISA after 72 h. **(B)** WT and *Il10*^-/-^ mice (n=18 per group, pooled 3 experiments) were immunized for EAE induction and received daily i.p. injections of PBS or rIFN-β. Clinical severity scores are shown. **(C)** Splenic WT or *Il27ra*^-/-^ naïve CD4^+^ T cells were co-cultured with WT or *Il27ra*^-/-^ APCs, activated with anti-CD3/28 Abs, with or without addition of rIFN-β into culturing media. GM-CSF concentrations in 72 h cell culture supernatants were measured by ELISA. **(D)** WT and *Il27ra*^-/-^ mice (n=6 per group), were immunized for EAE induction and treated with PBS or rIFN-β daily. Clinical severity score is shown. EAE experiments were conducted at least two times with similar outcomes. Data shown are mean ± SEM. For EAE, p-values were calculated using two-way ANOVA with Bonferroni’s multiple comparison correction. Parametric datasets were analyzed using unpaired Student’s *t*-test; *p < 0.05, **p < 0.01, ***p < 0.001, NS, not significant.

We next tested whether rIFN-β suppression of GM-CSF production by Th cells requires IL-10 by co-culturing WT or *Il10rb*^-/-^ naïve CD4^+^ T cells and APCs with addition of rIFN-β. rIFN-β had notably stronger suppressive effect on GM-CSF production by Th cells if they expressed IL-10R (~60% reduction), whereas IL-10 signaling in APCs resulted in modest suppressive effect on GM-CSF production by Th cells (~20% reduction) (**Figure 5A**). We next tested whether IL-10 is required for the suppressive effect of rIFN-β *in vivo*. WT and *Il10*^-/-^ mice were immunized for EAE induction and treated with rIFN-β daily. rIFN-β suppressed EAE in WT mice, but not in *Il10*^-/-^ mice (**Figure 5B**).

It has been shown that rIFN-β induces IL-27 *in vitro*, and that IL-27 induces IL-10 and suppresses GM-CSF expression (43). We therefore speculated that rIFN-β inhibits GM-CSF expression, and suppresses EAE by inducing IL-27 expression. However, rIFN-β suppressed both GM-CSF production and EAE independently of IL-27 signaling (**Figures 5C, D**). In addition, rIFN-β induced IL-10 production by BMDMs irrespective of IL-27 signaling (**Supplementary Figure 2E**). Taken together, these results show that rIFN-β suppresses GM-CSF production by Th cells and EAE *via* IL-10 signaling in Th cells, but independently of IL-27.

### rIFN-β Suppresses GM-CSF Expression in Th Cells by Reducing Secretion of IL-1β From Myeloid Cells

**Figure 6:**
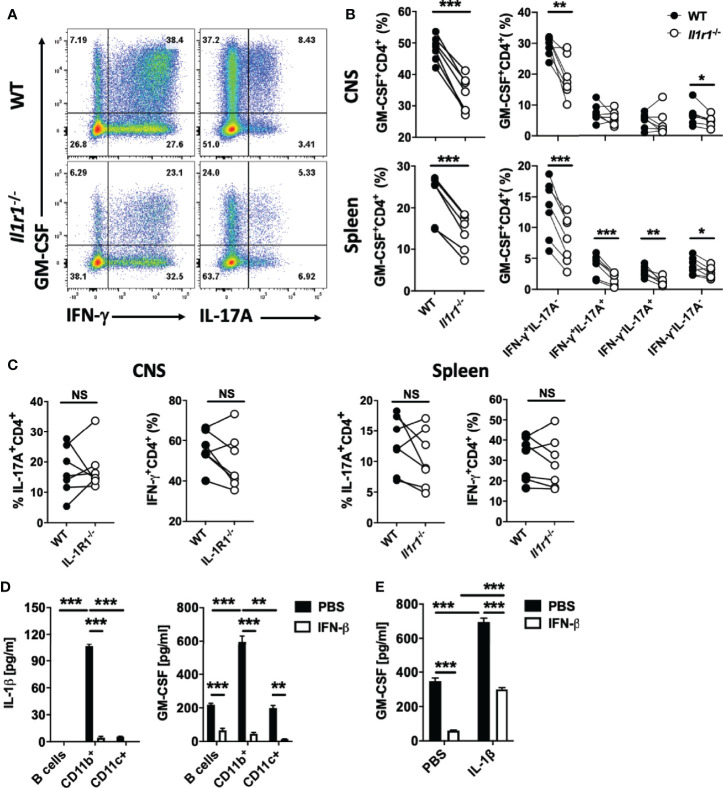
IL-1β induces GM-CSF expression in Th cells. Splenic WT (CD45.1) and *Il1r1*^-/-^ (CD45.2) CD4^+^ T cells in a 1:1 ratio (total 1 x 10^7^ cells) were co-transferred into recipient *Rag1*^-/-^ mice. Recipient mice (n=7) were immunized for EAE induction, and cells obtained from the spleen and CNS were analyzed at disease peak. **(A)** Representative flow cytometry dot plots showing GM-CSF, IFN-γ, and IL-17A expression by WT and *Il1r1*^-/-^ CD4^+^ T cells from the CNS of mice with EAE. **(B)** Proportions (%) of GM-CSF^+^, and different subsets of GM-CSF^+^CD4^+^ T cells in WT and *Il1r1*^-/-^ CD4^+^ T cells from the spleen and CNS of mice with EAE. **(C)** Proportions (%) of IFN-γ^+^, and IL-17A^+^ CD4^+^ T cells among WT and *Il1r1*^-/-^ CD4^+^ T cells from the spleen and CNS of mice with EAE. **(D)** Splenic naïve CD4^+^ T cells were co-cultured with FACS-sorted CD19^+^ (B cells), CD11b^+^, or CD11c^+^ cells from WT splenocytes, and activated with anti-CD3/28 Abs, with or without addition of rIFN-β into culturing media. IL-1β and GM-CSF concentrations in cell culture supernatants were measured by ELISA after 72 h of culturing. **(E)** Splenic naïve CD4^+^ T cells were co-cultured with CD11b^+^ cells and activated with anti-CD3/28 Abs, with or without addition of IL-1β (20 ng/ml) and rIFN-β into culturing media. GM-CSF concentrations in cell culture supernatants were measured by ELISA after 72 h of culturing. These experiments were conducted three times with similar outcomes. D**a**ta shown are mean ± SEM. P-values were calculated using paired Student’s *t*-test in **(B, C)**, and one-way ANOVA with Bonferroni’s multiple comparisons test in **(D, E)**; **p < 0.01, ***p < 0.001, NS, not significant.

rIFN-β suppressed expression of both IL-1α and IL-1β *in vitro* (**Supplementary Figures 5A–C**). IL-1α and IL-1β signal through the same receptor comprising IL-1R1 and IL-RAcP (44). IL-1β and IL-1R1 are required for the development of EAE, whereas IL-1α is not (45), leading us to focus on IL-1β in the rest of the study. rIFN-β abrogated IL-1β production in co-cultures of naïve CD4^+^ T cells and APCs by acting on APCs (**Supplementary Figure 5D**). Given that IL-1β induces GM-CSF expression in Th cells (26, 28), we hypothesized that rIFN-β suppresses their GM-CSF expression by inhibiting IL-1β expression by APCs. We confirmed that IL-1β induces GM-CSF production by Th cells *in vitro*, in a dose- dependent manner (**Supplementary Figures 5E, F**). Blocking or absence of IL-1R1 signaling reduced GM-CSF production by Th cells *in vitro* (**Supplementary Figures 5G, H**). To test whether IL-1β induces GM-CSF production by Th cells *in vivo*, we co-transferred total CD4^+^ T cells from WT (CD45.1) and *Il1r1*^-/-^ (CD45.2) mice into *Rag1*^-/-^ mice and immunized them for EAE induction; CD4^+^ T cells from the spleen and CNS were then analyzed at disease peak. The proportions of total GM-CSF^+^ *Il1r1*^-/-^ Th cells were lower than of WT Th cells, both in the spleen and CNS. Lack of IL-1R1 on CD4^+^ T cells reduced numbers of various GM-CSF^+^ Th cells (**Figures 6A, B**), without affecting overall numbers of Th1 and Th17 cells (**Figure 6C**). Even though GM-CSF expression by *Il1r1*^-/-^ Th cells was reduced, a proportion of these cells expressed GM-CSF, demonstrating that IL-1β is not absolutely required for GM-CSF production by Th cells. These results show that IL-1β induces GM-CSF expression in Th cells both *in vitro* and *in vivo*.

To identify cells that produce IL-1β, we co-cultured naïve CD4^+^ T cells with splenic CD11b^+^, CD11c^+^CD11b^-^ (DCs), and B cells of WT mice. CD11b^+^ cells produced by far the most IL-1β, and CD4^+^ T cells co-cultured with them secreted the most GM-CSF (**Figure 6D**). We next tested whether adding IL-1β in co-cultures of CD4^+^ T cells and CD11b^+^ cells would counteract the suppressive effect of rIFN-β on GM-CSF production. Exogenous IL-1β induced higher GM-CSF production by Th cells, but only partially ameliorated the suppression by rIFN-β (**Figure 6E**). These data indicate that the suppressive effect of rIFN-β on GM-CSF production by Th cells is mediated, in part, by suppression of IL-1β secretion from myeloid cells.

### rIFN-β Suppresses IL-1β Expression by CNS-Infiltrated Monocytes

**Figure 7:**
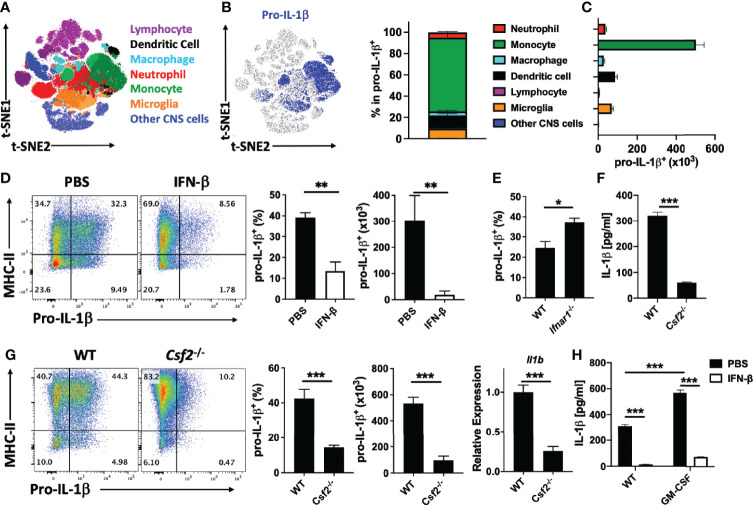
rIFN-β suppresses pro-IL-1β expression by the CNS monocytes. WT mice (n=5) were immunized for EAE induction, sacrificed at disease peak (day 15 p.i.), and mononuclear cells from the CNS analyzed by flow cytometry. **(A)** *t*-SNE plot showing seven populations of cells from CNS that were generated by PhenoGraph unbiased clustering. **(B)** Proportions (%) of CNS cell populations expressing pro-IL-1β. **(C)** Total numbers of pro-IL-1β-expressing cells from the CNS. **(D)** WT mice (n=4 per group) were immunized for EAE induction and treated daily with PBS or rIFN-β. Mice were sacrificed at disease peak (day 15 p.i.) and monocytes from the CNS analyzed by flow cytometry for pro-IL-1β expression. Representative flow cytometry dot plots showing MHC-II and pro-IL-1β expression by monocytes in the CNS of mice with EAE. Proportions (%) and total numbers of pro-IL-1β^+^ monocytes in the CNS of mice with EAE. **(E)** WT and *Ifnar1*^-/-^ mice (n=5 per group) were immunized for EAE induction, sacrificed at disease peak (day 15 p.i.), and monocytes from the CNS analyzed by flow cytometry for pro-IL-1β expression. Proportions (%) of pro-IL-1β^+^ monocytes from the CNS of mice with EAE are shown. **(F)** Splenic WT and *Csf2*^-/-^ naïve CD4^+^ T cells were co-cultured with WT APCs, activated with anti-CD3/28 Abs, and GM-CSF concentrations in cell culture supernatants measured by ELISA after 72 h of culturing. **(G)** WT and *Csf2*^-/-^ mice (n=5 per group) were immunized for EAE induction, sacrificed at disease peak (day 15 p.i.), and CNS infiltrated monocytes analyzed by flow cytometry for pro-IL-1β expression. Representative flow cytometry dot plots showing MHC-II and pro-IL-1β expression. Proportions (%) and total number of pro-IL-1β^+^ monocytes. *Il1b* expression by mononuclear cells from CNS of mice with EAE was determined by RT-PCR. **(H)** Splenic naïve CD4^+^ T cells were co-cultured with APCs and activated with anti-CD3/28 Abs with or without addition of GM-CSF (20 ng/ml) and rIFN-β. GM-CSF concentrations in cell culture supernatants were measured by ELISA after 72 h of culturing. These experiments were conducted three times with similar outcomes. Data shown are mean ± SEM. P-values were calculated using unpaired Student’s *t*-test in **(D**–**G)**, and one-way ANOVA with Bonferroni’s multiple comparisons test in **(H)**; *p < 0.05**p < 0.01, ***p < 0.001.

We next characterized pro-IL-1β-expressing cells from mice with EAE by flow cytometry. We found pro-IL-1β^+^ cells in the CNS, but not in the LN and spleen (data not shown). *t*-distributed Stochastic Neighbor Embedding (*t*-SNE) analysis and Phenograph unbiased clustering identified 7 populations among cells obtained from the CNS at EAE peak. Monocytes (CD11b^+^Ly6C^hi^Ly6G^-^CD45^hi^) were the predominant IL-1β^+^ population, followed by DCs (CD11c^+^MHCII^+^CD45^hi^) and microglia (CD11b^+^CD45^int^) (**Figures 7A–C**). We next tested whether rIFN-β treatment affects IL-1β expression by cells in the CNS of mice with EAE and found that treated mice had fewer IL-1β^+^ monocytes at EAE peak. (**Figure 7D**). It has been shown that rIFN-β suppresses MHC-II expression by APCs and limits Ag-presentation to Th cells (9); however; we did not find a significant decrease in MHC-II expression by myeloid cells from the CNS of mice with EAE (**Supplementary Figure 6A**). We also tested the role of endogenous IFN-β in regulation of IL-1β expression by CNS monocytes of WT and *Ifnar1*^-/-^ mice with EAE. Consistent with the effect of rIFN-β treatment, WT mice had fewer IL-1β^+^ monocytes than *Ifnar1*^-/-^ mice (**Figure 7E**). These data demonstrate that endogenously produced type I IFNs reduce expression of IL-1β by monocytes in the CNS of mice with EAE, and that treatment with rIFN-β further reduces its expression.

It has been shown that the encephalitogenic role of GM-CSF is due to its effects on monocytes (6), and we show here that they are the major source of IL-1β in the CNS of mice with EAE (**Figures 7A–C**). To determine to what extent GM-CSF production by Th cells impacts IL-1β secretion by myeloid cells, we co-cultured WT and *Csf2*^-/-^ CD4^+^ T cells with WT CD11b^+^ cells. We found much lower IL-1β concentrations in media from co-cultures with *Csf2*^-/-^ Th cells (**Figure 7F**). We then determined the role of GM-CSF in IL-1β expression in mice with EAE. As previously described (26), *Csf2*^-/-^ mice developed mild clinical EAE, with fewer immune cells in the CNS than in WT mice. *Csf2*^-/-^ mice had reduced frequencies and total numbers of IL-1β^+^ monocyte-derived cells in their CNS compared with WT mice, as well as diminished IL-1β expression by monocyte-derived cells (**Figure 7G**).

To determine if rIFN-β suppresses IL-1β production by myeloid cells *via* suppression of GM-CSF expression by Th cells, we added GM-CSF to the co-culture of WT CD4^+^ T cells and CD11b^+^ cells. Exogenous GM-CSF facilitated IL-1β production, but rIFN-β still efficiently suppressed it (**Figure 7H**). We also tested whether rIFN-β suppresses IL-1β production *via* IL-10 or IL-27 and found that these cytokines are disposable for the suppression (**Supplementary Figures 6B, C**). These data show that rIFN-β suppresses IL-1β secretion by myeloid cells *via* reduction in GM-CSF production by Th cells, and by an additional mechanism independent of IL-10 and IL-27.

### rIFN-β Suppresses IL-1β Expression in Myeloid Cells *via* Inhibition of GM-CSF/TNF/FASL Expression by Th Cells

**Figure 8:**
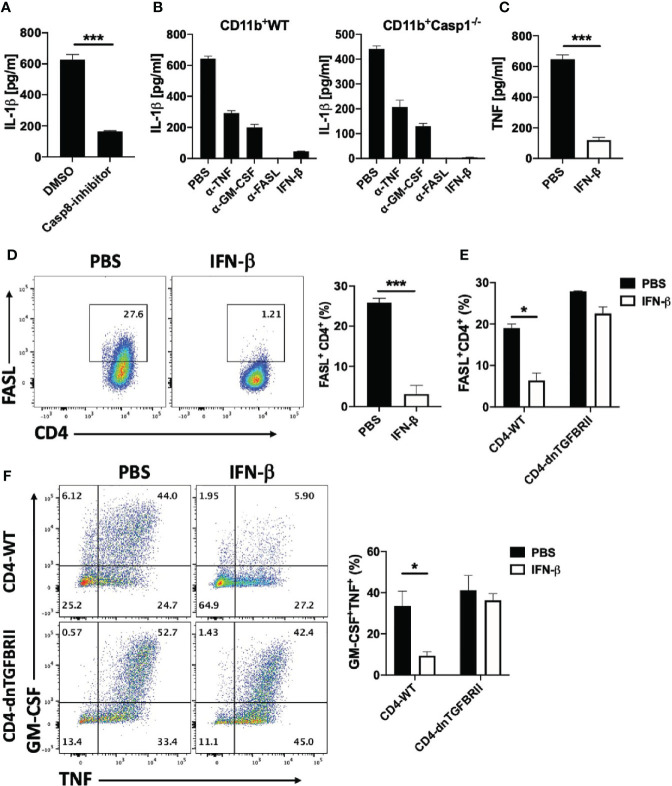
rIFN-β suppresses IL-1β expression in myeloid cells *via* inhibition of FASL expression by Th cells. Splenic naïve CD4^+^ T cells and CD11b^+^ cells were activated with anti-CD3/28 Abs with addition of caspase-8 inhibitor or DMSO into culturing media. **(A)** IL-1β concentrations in cell culture supernatants measured by ELISA after 72 h of culturing. **(B)** Splenic WT naïve CD4^+^ T cells were co-cultured with WT or *Casp1*^-/-^ CD11b^+^ cells and activated with anti-CD3/28 Abs, with or without addition of anti-TNF, anti-GM-CSF, anti-FASL Abs or rIFN-β into culturing media. IL-1β concentrations in cell culture supernatants were measured by ELISA after 72 h of culturing. **(C)** Splenic naïve CD4^+^ T cells and CD11b^+^ cells were co-cultured and activated with anti-CD3/28 Abs with or without addition of rIFN-β into culturing media. TNF concentrations in cell culture supernatants were measured by ELISA after 72 h of culturing. **(D)** Representative flow cytometry dot plots showing FASL expression by CD4^+^ T cells in **(C)** Proportions (%) of FASL^+^CD4^+^ T cells in **(C, E)** Splenic naïve CD4^+^ T cells from WT or CD4-dnTGFBRII mice were co-cultured with WT CD11b^+^ cells activated with anti-CD3/28 Abs, with or without addition of rIFN-β into culturing media. Proportions (%) of FASL^+^CD4^+^ T cells were analyzed by flow cytometry. **(F)** Representative flow cytometry dot plots showing GM-CSF and TNF expression by WT CD4^+^ and CD4-dnTGFBRII T cells in **(E)** Bar graph showing proportions (%) of GM-CSF^+^TNF^+^CD4^+^ T cells by WT CD4^+^ and CD4-dnTGFBRII T cells in **(E)** These experiments were conducted two **(A, B, E, F)** and three times **(C, D)** with similar outcomes. Data shown are mean ± SEM. P-values were calculated using unpaired Student’s *t-*test; *p < 0.05, ***p < 0.001.

A recent study has shown that Th cells induce IL-1β production in BM-derived DCs (BMDCs) *via* TNF and FASL (a role of GM-CSF was not tested); FASL/FAS signaling activated caspase-8, which cleaved pro-IL-1β induced by TNF (46). We first confirmed these findings by co-culturing naïve CD4^+^ T cells and CD11b cells in the presence of caspase-8 inhibitor IETD. Blocking caspase-8 suppressed IL-1β production by myeloid cells (**Figure 8A**), while lack of caspase-1 had a modest effect, and rIFN-β suppressed IL-1β production independently of caspase-1 (**Figure 8B**). We also compared the effect of GM-CSF, TNF and FASL on IL-1β production by myeloid cells. Blocking FASL abrogated IL-1β production by myeloid cells, whereas blocking TNF or GM-CSF had partial inhibitory effects (**Figure 8B**), and simultaneous blocking of GM-CSF and TNF also abrogated IL-1β production (**Supplementary Figure 6D**). These results indicate that GM-CSF, TNF and FASL are the major Th cell-derived stimuli for induction of IL-1β production by myeloid cells.

We have already shown that rIFN-β suppresses GM-CSF production by Th cells, and we next tested whether rIFN-β also suppresses TNF and FASL expression by Th cells. rIFN-β strongly suppressed both TNF secretion (**Figure 8C**) and FASL expression (**Figure 8D**) by Th cells in co-culture with CD11b^+^ cells. Among the cytokines that regulate FASL expression, TGF-β signaling in Th cells was required to suppress FASL expression by rIFN-β (**Figure 8E**), as well as to suppress GM-CSF and TNF expression (**Figure 8F**). In contrast, immunomodulatory molecules such as IL-10 and PD-L1 were not required for inhibitory effect of rIFN-β on FASL expression by Th cells (**Supplementary Figure 6E**). These results show that rIFN-β suppresses IL-1β production by myeloid cells by inhibiting the expression of GM-CSF, TNF and FASL expression by Th cells in a TGF-β-dependent manner.

## Discussion

Injectable rIFN-β is the first FDA-approved disease-modifying therapy for relapsing-remitting MS; however, the mechanism of its action is not well understood. Existent findings support the possibility that an important mechanism underlying the beneficial effects of rIFN-β in autoimmune neuroinflammation could be inhibition of GM-CSF production by Th cells. Here, we have expanded upon previous findings by elucidating this inhibitory mechanism in EAE.

It has been shown that IFNAR1 signaling in myeloid cells, triggered by endogenous IFNs, reduces EAE severity and lethality (9). We confirmed this observation, and extend it by identifying monocytes as a myeloid cell subset relevant to the beneficial effects of rIFN-β therapy in EAE. Our data showing that the effects of rIFN-β on GM-CSF production by Th cells is indirect, *via* myeloid cells, is also consistent with a previous finding that IFNAR1 signaling in lymphocytes is irrelevant for the role of endogenous type-I IFNs in EAE (9). rIFN-β treatment induced IL-10 expression by monocytes, macrophages, DCs, and Th cells in the CNS of mice with EAE, and IL-10 was required for suppressive effects of rIFN-β. Even though it is widely accepted that rIFN-β-induced IL-10 mediates the suppressive effect of rIFN-β in EAE (and MS), to the best of our knowledge, our data shown here directly validate this assumption for the first time. Given that rIFN-β induces IL-27 expression in APCs activated with TLR agonist (43, 47, 48), and that IL-27 potently induces IL-10 expression and suppresses GM-CSF production by Th cells (49–51), it has been inferred that rIFN-β induces IL-10 expression *via* IL-27, and that rIFN-β therapy relies on IFN-β/IL-27/IL-10 axis (43, 47, 48). However, our findings refute this widely held assumption, because rIFN-β induced IL-10 production independently of IL-27, and, most important, rIFN-β treatment suppressed EAE in the absence of IL-27 signaling, as we have previously shown (47). In addition, rIFN-β suppressed GM-CSF expression by Th cells independently of IL-27. Our findings clearly show that IL-27 is not an important mediator in rIFN-β therapy in EAE, and possibly not in MS.

Recent characterization of monocytes from the CNS of mice with EAE revealed a new subset with high expression of type-I IFN-related genes, but the role of these monocytes has not been elucidated (52). We show that in the CNS of mice with EAE, rIFN-β treatment induces similar monocyte subset with pDC-like phenotype, expressing IFN-β, type-I IFN-related genes, PDCA-1, and Siglec-H. pDCs are known as the major source of IFN-β, but monocytes/macrophages can also produce IFN-β during viral infections and in EAE (53–56). Given that pDCs have an immunoregulatory role in EAE (57, 58), it is likely that pDC-like monocytes induced by rIFN-β have similar anti-inflammatory traits that contribute to EAE suppression. It has been shown that during chronic infection, IFN-α/β from macrophages and DCs induced IL-10 production in them to suppress T cell response (59, 60). It is therefore tempting to speculate that rIFN-β boosts production of IFN-β by monocytes to enhance secretion of IL-10, and amplify other immunoregulatory mechanisms as well. It remains to be elucidated to what extent endogenous IFN-β production contributes to disease suppression by the treatment with rIFN-β.

IL-1β, which is required for EAE development (45), was initially associated with the development of Th17 cells (61), but more recent studies emphasize its role in promoting GM-CSF expression by Th cells (26, 28). Our results confirmed that IL-1β signaling is not necessary for Th17 development, but rather for GM-CSF expression by Th cells. Even though IL-1β has a strong GM-CSF-inducing effect, GM-CSF expression by Th cells was not abrogated without IL-1β signaling, indicating that other cytokines, such as IL-2, IL-7, and IL-23 (28), also induce GM-CSF expression. The relationship between IL-1β and GM-CSF is reciprocal, as GM-CSF induces IL-1β expression in myeloid cells and the lack of GM-CSF drastically reduces IL-1β expression by monocytes in the CNS of mice with EAE. Hence, IL-1β and GM-CSF form a positive feed-back loop between APCs and Th cells that potentiates their effector functions in inflammation. Myeloid cells can secrete IL-1β through at least two pathways. During infection, activation of PRRs leads to cleavage of pro-IL-1β by inflammasome component NLRP3 and caspase-1 (62). In contrast, Th cell-induced cleavage of pro-IL-1β in myeloid cells relies on activation of caspase-8, independent of inflammasome. In this pathway, TNF from Th cells induces expression of pro-IL-1β in myeloid cells, and simultaneous engagement of FASL/FAS between Th cells and myeloid cells activates caspase-8 to cleave pro-IL-1β and secrete bioactive IL-1β (46). We confirmed that Th cells induce IL-1β expression by myeloid cells by TNF and FASL, but, we also identified GM-CSF as an additional important inducer of caspase-8/IL-1β pathway. Hence, a complete pathway encompasses GM-CSF/TNF/FASL expressed by Th cells. This view is supported by findings that mice lacking TNF signaling develop only mild EAE (63–65), and that mice lacking GM-CSF or FAS/FASL signaling are resistant to EAE (16, 66, 67).

IFN-β acted on myeloid cells to induce IL-10 and TGF-β production. IL-10 acted on Th cells to suppress their GM-CSF production, but did not affect FASL and IL-1β expression by myeloid cells. This is in contrast to the previous report that PRR-activated myeloid cells upregulate IL-10 in response to rIFN-β, and that IL-10 in an autocrine manner suppresses IL-1β production (62). The difference in findings could be due to dissimilar experimental designs, as we relied on T cell activation of myeloid cells. Moreover, TGF-β signaling in Th cells was required for suppression of their FASL, GM-CSF and TNF expression upon treatment of Th cell-APC co-cultures with rIFN-β. Given that myeloid cells lacking TGF-β signaling are not readily available, and that mice lacking TGF-β signaling in CD4^+^ T cells are resistant to EAE (68), it remains to be determined if TGF-β signaling in myeloid cells contributes to the suppression of their IL-1β production, and if TGF-β plays a role in the effects of rIFN-β in EAE.

It has been shown that rIFN-β induces PD-L1 expression by human monocytes *in vitro*, and that MS patients treated with rIFN-β have increased levels of PD-L1 mRNA (69). Given that PD-L1 strongly inhibits activation, proliferation and cytokine secretion of CD4^+^ T cells, it is possible that an important mechanism of EAE suppression by rIFN-β relies on induction of PD-L1 expression by monocytes. Another study found that rIFN-β therapy results in upregulation of both co-stimulatory and regulatory molecules (including PD-L1) on monocytes of MS patients, but upregulation of PD-L2, not of PD-L1, correlated with beneficial outcomes of rIFN-β therapy (70). Similar upregulation of PD-L1 upon rIFN-β treatment has been observed in other studies with MS patients and mice with EAE (71, 72), but its functional relevance in disease suppression remains to be determined. We tested a role of PD-L1 in the suppressive effect of rIFN-β on FASL expression by Th cells *in vitro*, but rIFN-β suppressed FASL expression by Th cells independent of PD-L1 expression by myeloid cells. This, however, does not exclude a possibility that rIFN-β-induced PD-L1 suppresses EAE *via* other pathway(s).

In summary, we show that rIFN-β suppresses CNS inflammation by acting on monocytes to induce an anti-inflammatory phenotype in them. These monocytes ameliorate CNS inflammation by producing IL-10 and possibly TGF-β to suppress the encephalitogenic features of Th cells, namely their GM-CSF, TNF, and FASL expression, resulting in inhibition of a proinflammatory feedback loop between Th cells and APCs (**Supplementary Figure 7**).

## Data Availability Statement

The datasets presented in this study can be found in online repositories. The names of the repository/repositories and accession number(s) can be found below: (https://www.ncbi.nlm.nih.gov/geo/query/acc.cgi?acc=GSE169670).

## Ethics Statement

The animal study was reviewed and approved by Institutional Animal Care and Use Committee of Thomas Jefferson University.

## Author Contributions

JR designed and performed experiments, wrote the manuscript, and evaluated and interpreted the data. GC, LI, RT, AB, CM-S, EM, PP, WZ, and DX performed experiments and edited the manuscript. AE performed RNA-seq analysis and data interpretation. PF, LS, and G-XZ revised the manuscript. AR supervised and financed the studies. BC supervised the study, designed the experiments, interpreted the data, and wrote the manuscript. All authors contributed to the article and approved the submitted version.

## Funding

This study was supported by the National Institutes of Health, USA (AI146796-01 to BC and 5R01AI124386 to AR).

## Conflict of Interest

The authors declare that the research was conducted in the absence of any commercial or financial relationships that could be construed as a potential conflict of interest.

## References

[1] Lublin FD Reingold SC Cohen JA Cutter GR Sorensen PS Thompson AJ. Defining the Clinical Course of Multiple Sclerosis: The 2013 Revisions. Neurology (2014) 83(3):278–86. 10.1212/WNL.0000000000000560 PMC4117366 24871874

[2] Giles DA Duncker PC Wilkinson NM Washnock-Schmid JM Segal BM. CNS-Resident Classical DCs Play a Critical Role in CNS Autoimmune Disease. J Clin Invest (2018) 128(12):5322–34. 10.1172/JCI123708 PMC6264723 30226829

[3] Mundt S Mrdjen D Utz SG Greter M Schreiner B Becher B. Conventional DCs Sample and Present Myelin Antigens in the Healthy CNS and Allow Parenchymal T Cell Entry to Initiate Neuroinflammation. Sci Immunol (2019) 4(31). 10.1126/sciimmunol.aau8380 30679199

[4] Kivisakk P Mahad DJ Callahan MK Sikora K Trebst C Tucky B. Expression of CCR7 in Multiple Sclerosis: Implications for CNS Immunity. Ann Neurol (2004) 55(5):627–38. 10.1002/ana.20049 15122702

[5] Serafini B Rosicarelli B Magliozzi R Stigliano E Capello E Mancardi GL. Dendritic Cells in Multiple Sclerosis Lesions: Maturation Stage, Myelin Uptake, and Interaction With Proliferating T Cells. J Neuropathol Exp Neurol (2006) 65(2):124–41. 10.1093/jnen/65.2.124 16462204

[6] Croxford AL Lanzinger M Hartmann FJ Schreiner B Mair F Pelczar P. The Cytokine Gm-Csf Drives the Inflammatory Signature of CCR2+ Monocytes and Licenses Autoimmunity. Immunity (2015) 43(3):502–14. 10.1016/j.immuni.2015.08.010 26341401

[7] Croxford AL Spath S Becher B. Gm-CSF in Neuroinflammation: Licensing Myeloid Cells for Tissue Damage. Trends Immunol (2015) 36(10):651–62. 10.1016/j.it.2015.08.004 26431942

[8] Ivashkiv LB Donlin LT. Regulation of Type I Interferon Responses. Nat Rev Immunol (2014) 14(1):36–49. 10.1038/nri3581 24362405 PMC4084561

[9] Prinz M Schmidt H Mildner A Knobeloch KP Hanisch UK Raasch J. Distinct and Nonredundant In Vivo Functions of IFNAR on Myeloid Cells Limit Autoimmunity in the Central Nervous System. Immunity (2008) 28(5):675–86. 10.1016/j.immuni.2008.03.011 18424188

[10] Axtell RC de Jong BA Boniface K van der Voort LF Bhat R De Sarno P. T Helper Type 1 and 17 Cells Determine Efficacy of Interferon-Beta in Multiple Sclerosis and Experimental Encephalomyelitis. Nat Med (2010) 16(4):406–12. 10.1038/nm.2110 PMC3042885 20348925

[11] Lublin F. History of Modern Multiple Sclerosis Therapy. J Neurol (2005) 252(Suppl 3):iii3–9. 10.1007/s00415-005-2010-6 16170498

[12] Interferon Beta-1b Is Effective in Relapsing-Remitting Multiple Sclerosis. I. Clinical Results of a Multicenter, Randomized, Double-Blind, Placebo-Controlled Trial. The IFNB Multiple Sclerosis Study Group. Neurology (1993) 43(4):655–61. 10.1212/WNL.43.4.655 8469318

[13] van Holten J Plater-Zyberk C Tak PP. Interferon-Beta for Treatment of Rheumatoid Arthritis? Arthritis Res (2002) 4(6):346–52. 10.1186/ar598 PMC153843 12453310

[14] Markowitz CE. Interferon-Beta: Mechanism of Action and Dosing Issues. Neurology (2007) 68(24 Suppl 4):S8–11. 10.1212/01.wnl.0000277703.74115.d2 17562848

[15] Axtell RC Raman C Steinman L. Interferon-Beta Exacerbates Th17-mediated Inflammatory Disease. Trends Immunol (2011) 32(6):272–7. 10.1016/j.it.2011.03.008 PMC5414634 21530402

[16] McQualter JL Darwiche R Ewing C Onuki M Kay TW Hamilton JA. Granulocyte Macrophage Colony-Stimulating Factor: A New Putative Therapeutic Target in Multiple Sclerosis. J Exp Med (2001) 194(7):873–82. 10.1084/jem.194.7.873 PMC2193476 11581310

[17] Ponomarev ED Shriver LP Maresz K Pedras-Vasconcelos J Verthelyi D Dittel BN. Gm-CSF Production by Autoreactive T Cells Is Required for the Activation of Microglial Cells and the Onset of Experimental Autoimmune Encephalomyelitis. J Immunol (2007) 178(1):39–48. 10.4049/jimmunol.178.1.39 17182538

[18] Ifergan I Davidson TS Kebir H Xu D Palacios-Macapagal D Cann J. Targeting the GM-CSF Receptor for the Treatment of CNS Autoimmunity. J Autoimmun (2017) 84:1–11. 10.1016/j.jaut.2017.06.005 28641926 PMC5647260

[19] Constantinescu CS Asher A Fryze W Kozubski W Wagner F Aram J. Randomized Phase 1B Trial of MOR103, a Human Antibody to GM-CSF, in Multiple Sclerosis. Neurol Neuroimmunol Neuroinflamm (2015) 2(4):e117. 10.1212/NXI.0000000000000117 26185773 PMC4442097

[20] Baldwin GC Gasson JC Kaufman SE Quan SG Williams RE Avalos BR. Nonhematopoietic Tumor Cells Express Functional GM-CSF Receptors. Blood (1989) 73(4):1033–7. 10.1182/blood.V73.4.1033.bloodjournal7341033 2537665

[21] Dedhar S Gaboury L Galloway P Eaves C. Human Granulocyte-Macrophage Colony-Stimulating Factor is a Growth Factor Active on a Variety of Cell Types of Nonhemopoietic Origin. Proc Natl Acad Sci USA (1988) 85(23):9253–7. 10.1073/pnas.85.23.9253 PMC282717 3057504

[22] Bussolino F Wang JM Defilippi P Turrini F Sanavio F Edgell CJ. Granulocyte- and Granulocyte-Macrophage-Colony Stimulating Factors Induce Human Endothelial Cells to Migrate and Proliferate. Nature (1989) 337(6206):471–3. 10.1038/337471a0 2464767

[23] Bussolino F Ziche M Wang JM Alessi D Morbidelli L Cremona O. In Vitro and In Vivo Activation of Endothelial Cells by Colony-Stimulating Factors. J Clin Invest (1991) 87(3):986–95. 10.1172/JCI115107 PMC329891 1705569

[24] Rivas CI Vera JC Delgado-Lopez F Heaney ML Guaiquil VH Zhang RH. Expression of Granulocyte-Macrophage Colony-Stimulating Factor Receptors in Human Prostate Cancer. Blood (1998) 91(3):1037–43. 10.1182/blood.V91.3.1037 9446667

[25] Imitola J Rasouli J Watanabe F Mahajan K Sharan AD Ciric B. Elevated Expression of Granulocyte-Macrophage Colony-Stimulating Factor Receptor in Multiple Sclerosis Lesions. J Neuroimmunol (2018) 317:45–54. 10.1016/j.jneuroim.2017.12.017 29290406 PMC5935005

[26] El-Behi M Ciric B Dai H Yan Y Cullimore M Safavi F. The Encephalitogenicity of T(H)17 Cells Is Dependent on IL-1- and IL-23-induced Production of the Cytokine GM-CSF. Nat Immunol (2011) 12(6):568–75. 10.1038/ni.2031 PMC3116521 21516111

[27] Codarri L Gyulveszi G Tosevski V Hesske L Fontana A Magnenat L. Rorgammat Drives Production of the Cytokine GM-CSF in Helper T Cells, Which is Essential for the Effector Phase of Autoimmune Neuroinflammation. Nat Immunol (2011) 12(6):560–7. 10.1038/ni.2027 21516112

[28] Rasouli J Casella G Yoshimura S Zhang W Xiao D Garifallou J. A Distinct GM-CSF(+) T Helper Cell Subset Requires T-bet to Adopt a TH1 Phenotype and Promote Neuroinflammation. Sci Immunol (2020) 5(52). 10.1126/sciimmunol.aba9953 33097590

[29] Rasouli J Ciric B Imitola J Gonnella P Hwang D Mahajan K. Expression of GM-CSF in T Cells Is Increased in Multiple Sclerosis and Suppressed by IFN-beta Therapy. J Immunol (2015) 194(11):5085–93. 10.4049/jimmunol.1403243 PMC4433790 25917097

[30] Hartmann FJ Khademi M Aram J Ammann S Kockum I Constantinescu C. Multiple Sclerosis-Associated IL2RA Polymorphism Controls GM-CSF Production in Human TH Cells. Nat Commun (2014) 5:5056. 10.1038/ncomms6056 25278028

[31] Galli E Hartmann FJ Schreiner B Ingelfinger F Arvaniti E Diebold M. Gm-CSF and CXCR4 Define a T Helper Cell Signature in Multiple Sclerosis. Nat Med (2019) 25(8):1290–300. 10.1038/s41591-019-0521-4 PMC6689469 31332391

[32] Noster R Riedel R Mashreghi MF Radbruch H Harms L Haftmann C. Il-17 and GM-CSF Expression are Antagonistically Regulated by Human T Helper Cells. Sci Transl Med (2014) 6(241):241ra80. 10.1126/scitranslmed.3008706 24944195

[33] Casella G Garzetti L Gatta AT Finardi A Maiorino C Ruffini F. IL4 Induces IL6-Producing M2 Macrophages Associated to Inhibition of Neuroinflammation In Vitro and In Vivo. J Neuroinflamm (2016) 13(1):139. 10.1186/s12974-016-0596-5 PMC4895901 27266518

[34] Thome R Moore JN Mari ER Rasouli J Hwang D Yoshimura S. Induction of Peripheral Tolerance in Ongoing Autoimmune Inflammation Requires Interleukin 27 Signaling in Dendritic Cells. Front Immunol (2017) 8:1392. 10.3389/fimmu.2017.01392 29163476 PMC5663690

[35] Subramanian A Tamayo P Mootha VK Mukherjee S Ebert BL Gillette MA. Gene Set Enrichment Analysis: A Knowledge-Based Approach for Interpreting Genome-Wide Expression Profiles. Proc Natl Acad Sci USA (2005) 102(43):15545–50. 10.1073/pnas.0506580102 PMC1239896 16199517

[36] Liberzon A Subramanian A Pinchback R Thorvaldsdottir H Tamayo P Mesirov JP. Molecular Signatures Database (MsigDB) 3.0. Bioinformatics (2011) 27(12):1739–40. 10.1093/bioinformatics/btr260 PMC3106198 21546393

[37] Villani AC Satija R Reynolds G Sarkizova S Shekhar K Fletcher J. Single-Cell RNA-seq Reveals New Types of Human Blood Dendritic Cells, Monocytes, and Progenitors. Science (2017) 356(6335). 10.1126/science.aah4573 PMC5775029 28428369

[38] Rodrigues PF Alberti-Servera L Eremin A Grajales-Reyes GE Ivanek R Tussiwand R. Distinct Progenitor Lineages Contribute to the Heterogeneity of Plasmacytoid Dendritic Cells. Nat Immunol (2018) 19(7):711–22. 10.1038/s41590-018-0136-9 PMC7614340 29925996

[39] Izikson L Klein RS Charo IF Weiner HL Luster AD. Resistance to Experimental Autoimmune Encephalomyelitis in Mice Lacking the CC Chemokine Receptor (CCR)2. J Exp Med (2000) 192(7):1075–80. 10.1084/jem.192.7.1075 PMC2193310 11015448

[40] Boring L Gosling J Chensue SW Kunkel SL Farese RV Jr Broxmeyer HE. Impaired Monocyte Migration and Reduced Type 1 (Th1) Cytokine Responses in C-C Chemokine Receptor 2 Knockout Mice. J Clin Invest (1997) 100(10):2552–61. 10.1172/JCI119798 PMC508456 9366570

[41] Honda K Ohba Y Yanai H Negishi H Mizutani T Takaoka A. Spatiotemporal Regulation of MyD88-IRF-7 Signalling for Robust Type-I Interferon Induction. Nature (2005) 434(7036):1035–40. 10.1038/nature03547 15815647

[42] Perez OA Yeung ST Vera-Licona P Romagnoli PA Samji T Ural BB. CD169(+) Macrophages Orchestrate Innate Immune Responses by Regulating Bacterial Localization in the Spleen. Sci Immunol (2017) 2(16). 10.1126/sciimmunol.aah5520 PMC5969998 28986418

[43] Sweeney CM Lonergan R Basdeo SA Kinsella K Dungan LS Higgins SC. Il-27 Mediates the Response to IFN-beta Therapy in Multiple Sclerosis Patients by Inhibiting Th17 Cells. Brain Behav Immun (2011) 25(6):1170–81. 10.1016/j.bbi.2011.03.007 21420486

[44] Dinarello CA. Immunological and Inflammatory Functions of the Interleukin-1 Family. Annu Rev Immunol (2009) 27:519–50. 10.1146/annurev.immunol.021908.132612 19302047

[45] Levesque SA Pare A Mailhot B Bellver-Landete V Kebir H Lecuyer MA. Myeloid Cell Transmigration Across the CNS Vasculature Triggers IL-1beta-Driven Neuroinflammation During Autoimmune Encephalomyelitis in Mice. J Exp Med (2016) 213(6):929–49. 10.1084/jem.20151437 PMC4886360 27139491

[46] Jain A Irizarry-Caro RA McDaniel MM Chawla AS Carroll KR Overcast GR. T Cells Instruct Myeloid Cells to Produce Inflammasome-Independent IL-1beta and Cause Autoimmunity. Nat Immunol (2020) 21(1):65–74. 10.1038/s41590-019-0559-y 31848486 PMC6927526

[47] Fitzgerald DC Fonseca-Kelly Z Cullimore ML Safabakhsh P Saris CJ Zhang GX. Independent and Interdependent Immunoregulatory Effects of IL-27, IFN-Beta, and IL-10 in the Suppression of Human Th17 Cells and Murine Experimental Autoimmune Encephalomyelitis. J Immunol (2013) 190(7):3225–34. 10.4049/jimmunol.1200141 PMC5027611 23455508

[48] Guo B Chang EY Cheng G. The Type I IFN Induction Pathway Constrains Th17-mediated Autoimmune Inflammation in Mice. J Clin Invest (2008) 118(5):1680–90. 10.1172/JCI33342 PMC2276397 18382764

[49] Awasthi A Carrier Y Peron JP Bettelli E Kamanaka M Flavell RA. A Dominant Function for Interleukin 27 in Generating Interleukin 10-Producing Anti-Inflammatory T Cells. Nat Immunol (2007) 8(12):1380–9. 10.1038/ni1541 17994022

[50] Fitzgerald DC Zhang GX El-Behi M Fonseca-Kelly Z Li H Yu S. Suppression of Autoimmune Inflammation of the Central Nervous System by Interleukin 10 Secreted by Interleukin 27-Stimulated T Cells. Nat Immunol (2007) 8(12):1372–9. 10.1038/ni1540 17994023

[51] Young A Linehan E Hams E O’Hara Hall AC McClurg A Johnston JA. Cutting Edge: Suppression of GM-CSF Expression in Murine and Human T Cells by IL-27. J Immunol (2012) 189(5):2079–83. 10.4049/jimmunol.1200131 PMC3424384 22837488

[52] Giladi A Wagner LK Li H Dorr D Medaglia C Paul F. Cxcl10(+) Monocytes Define a Pathogenic Subset in the Central Nervous System During Autoimmune Neuroinflammation. Nat Immunol (2020) 21(5):525–34. 10.1038/s41590-020-0661-1 32313246

[53] Ali S Mann-Nuttel R Schulze A Richter L Alferink J Scheu S. Sources of Type I Interferons in Infectious Immunity: Plasmacytoid Dendritic Cells Not Always in the Driver’s Seat. Front Immunol (2019) 10:778. 10.3389/fimmu.2019.00778 31031767 PMC6473462

[54] McNab F Mayer-Barber K Sher A Wack A O’Garra A. Type I Interferons in Infectious Disease. Nat Rev Immunol (2015) 15(2):87–103. 10.1038/nri3787 25614319 PMC7162685

[55] Xu RH Wong EB Rubio D Roscoe F Ma X Nair S. Sequential Activation of Two Pathogen-Sensing Pathways Required for Type I Interferon Expression and Resistance to an Acute Dna Virus Infection. Immunity (2015) 43(6):1148–59. 10.1016/j.immuni.2015.11.015 PMC4684903 26682986

[56] Kocur M Schneider R Pulm AK Bauer J Kropp S Gliem M. Ifnbeta Secreted by Microglia Mediates Clearance of Myelin Debris in CNS Autoimmunity. Acta Neuropathol Commun (2015) 3:20. 10.1186/s40478-015-0192-4 25853624 PMC4383054

[57] Bailey-Bucktrout SL Caulkins SC Goings G Fischer JA Dzionek A Miller SD. Cutting Edge: Central Nervous System Plasmacytoid Dendritic Cells Regulate the Severity of Relapsing Experimental Autoimmune Encephalomyelitis. J Immunol (2008) 180(10):6457–61. 10.4049/jimmunol.180.10.6457 PMC2846244 18453561

[58] Duraes FV Lippens C Steinbach K Dubrot J Brighouse D Bendriss-Vermare N. pDC Therapy Induces Recovery From EAE by Recruiting Endogenous pDC to Sites of CNS Inflammation. J Autoimmun (2016) 67:8–18. 10.1016/j.jaut.2015.08.014 26341385 PMC4758828

[59] Ng CT Oldstone MB. Infected CD8alpha- Dendritic Cells are the Predominant Source of IL-10 During Establishment of Persistent Viral Infection. Proc Natl Acad Sci USA (2012) 109(35):14116–21. 10.1073/pnas.1211910109 PMC3435180 22893686

[60] McNab FW Ewbank J Howes A Moreira-Teixeira L Martirosyan A Ghilardi N. Type I IFN Induces IL-10 Production in an IL-27-Independent Manner and Blocks Responsiveness to IFN-gamma for Production of IL-12 and Bacterial Killing in Mycobacterium Tuberculosis-Infected Macrophages. J Immunol (2014) 193(7):3600–12. 10.4049/jimmunol.1401088 PMC4170673 25187652

[61] Veldhoen M Hocking RJ Atkins CJ Locksley RM Stockinger B. Tgfbeta in the Context of an Inflammatory Cytokine Milieu Supports De Novo Differentiation of IL-17-Producing T Cells. Immunity (2006) 24(2):179–89. 10.1016/j.immuni.2006.01.001 16473830

[62] Guarda G Braun M Staehli F Tardivel A Mattmann C Forster I. Type I Interferon Inhibits Interleukin-1 Production and Inflammasome Activation. Immunity (2011) 34(2):213–23. 10.1016/j.immuni.2011.02.006 21349431

[63] Kassiotis G Pasparakis M Kollias G Probert L. TNF Accelerates the Onset But Does Not Alter the Incidence and Severity of Myelin Basic Protein-Induced Experimental Autoimmune Encephalomyelitis. Eur J Immunol (1999) 29(3):774–80. 10.1002/(SICI)1521-4141(199903)29:03<774::AID-IMMU774>3.0.CO;2-T 10092079

[64] Schiffenbauer J Streit WJ Butfiloski E LaBow M Edwards C 3rd Moldawer LL. The Induction of EAE is Only Partially Dependent on TNF Receptor Signaling But Requires the IL-1 Type I Receptor. Clin Immunol (2000) 95(2):117–23. 10.1006/clim.2000.4851 10779405

[65] Kruglov AA Lampropoulou V Fillatreau S Nedospasov SA. Pathogenic and Protective Functions of TNF in Neuroinflammation are Defined by Its Expression in T Lymphocytes and Myeloid Cells. J Immunol (2011) 187(11):5660–70. 10.4049/jimmunol.1100663 22058414

[66] Sabelko KA Kelly KA Nahm MH Cross AH Russell JH. Fas and Fas Ligand Enhance the Pathogenesis of Experimental Allergic Encephalomyelitis, But Are Not Essential for Immune Privilege in the Central Nervous System. J Immunol (1997) 159(7):3096–9. 9317103

[67] Waldner H Sobel RA Howard E Kuchroo VK. Fas- and FasL-deficient Mice are Resistant to Induction of Autoimmune Encephalomyelitis. J Immunol (1997) 159(7):3100–3. 9317104

[68] Veldhoen M Hocking RJ Flavell RA Stockinger B. Signals Mediated by Transforming Growth Factor-Beta Initiate Autoimmune Encephalomyelitis, But Chronic Inflammation Is Needed to Sustain Disease. Nat Immunol (2006) 7(11):1151–6. 10.1038/ni1391 16998492

[69] Schreiner B Mitsdoerffer M Kieseier BC Chen L Hartung HP Weller M. Interferon-Beta Enhances Monocyte and Dendritic Cell Expression of B7-H1 (Pd-L1), a Strong Inhibitor of Autologous T-Cell Activation: Relevance for the Immune Modulatory Effect in Multiple Sclerosis. J Neuroimmunol (2004) 155(1-2):172–82. 10.1016/j.jneuroim.2004.06.013 15342209

[70] Wiesemann E Deb M Trebst C Hemmer B Stangel M Windhagen A. Effects of Interferon-Beta on Co-Signaling Molecules: Upregulation of CD40, CD86 and PD-L2 on Monocytes in Relation to Clinical Response to Interferon-Beta Treatment in Patients With Multiple Sclerosis. Mult Scler (2008) 14(2):166–76. 10.1177/1352458507081342 17942524

[71] Feng X Bao R Li L Deisenhammer F Arnason BGW Reder AT. Interferon-Beta Corrects Massive Gene Dysregulation in Multiple Sclerosis: Short-term and Long-Term Effects on Immune Regulation and Neuroprotection. EBioMedicine (2019) 49:269–83. 10.1016/j.ebiom.2019.09.059 PMC6945282 31648992

[72] Harari D Kuhn N Abramovich R Sasson K Zozulya AL Smith P. Enhanced In Vivo Efficacy of a Type I Interferon Superagonist With Extended Plasma Half-Life in a Mouse Model of Multiple Sclerosis. J Biol Chem (2014) 289(42):29014–29. 10.1074/jbc.M114.602474 PMC4200257 25193661

