# Supplementary material for: IFN-β Acts on Monocytes to Ameliorate CNS Autoimmunity by Inhibiting Proinflammatory Cross-Talk Between Monocytes and Th Cells
Source: Front Immunol. 2021 Jun 4;12:679498. doi: 10.3389/fimmu.2021.679498 (PMC8213026; doi:10.3389/fimmu.2021.679498)
Supplement: Supplementary file 1 [file DataSheet_1.pdf]

IFN-β acts on monocytes to ameliorate CNS autoimmunity by inhibiting proinflammatory cross-talk between monocytes and Th cells

Javad Rasouli[1], Giacomo Casella[1], Larissa L. W. Ishikawa[1], Rodolfo Thome[1], Alexandra Boehm[1], Adam Ertel[3], Carolina R. Melo-Silva[2], Elisabeth R. Mari[1], Patrizia Porazzi[3], Weifeng Zhang[1], Dan Xiao[1], Luis J. Sigal[2], Paolo Fortina[3,4], Guang-Xian Zhang[1], Abdolmohamad Rostami[1], Bogoljub Ciric[1*].

[1]Department of Neurology, Thomas Jefferson University, Philadelphia, PA, USA
[2]Department of Microbiology and Immunology, Thomas Jefferson University, Philadelphia, PA, USA.
[3]Sidney Kimmel Cancer Center, Department of Cancer Biology, Thomas Jefferson University, Philadelphia, PA, USA.
[4]Department of Translation and Precision Medicine, Sapienza University, Rome, Italy

*Corresponding author:
B. Ciric, Bogoljub.Ciric@jefferson.edu
Department of Neurology, Jefferson Hospital for Neuroscience, Thomas Jefferson University, 900 Walnut Street, Suite 300, Philadelphia, PA 19107

SUPPLEMENTARY FIGURES:

Supplementary Figure 1

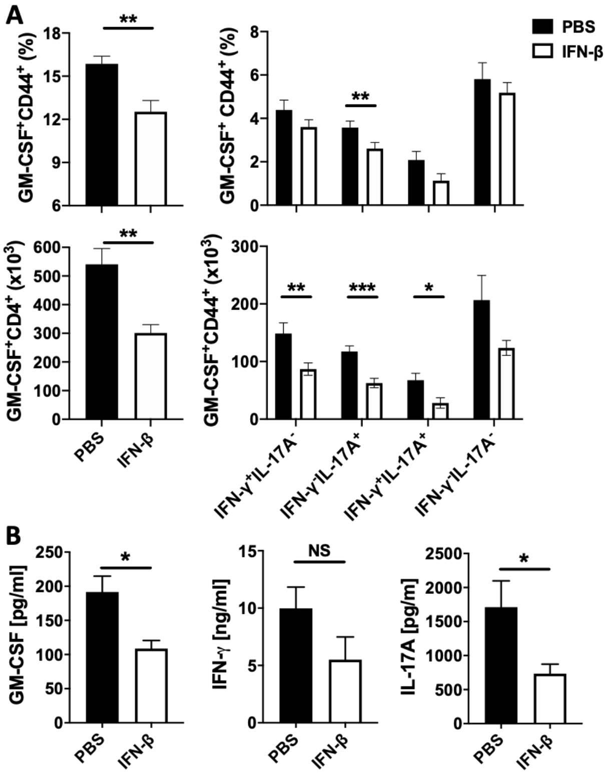

Supplementary Figure 1. rIFN-β decreases the numbers of GM-CSF-producing Th cells in the spleen of mice with EAE. Mice (n=10 per group) were immunized for EAE induction, treated daily with PBS or rIFN-β, sacrificed at day 8 p.i., and $CD4^+$ T cells from spleen analyzed by flow cytometry. **A)** Proportions and total numbers of GM-CSF^+, GM-CSF^+IFN-γ^+, and GM-CSF^+IL-$17A^+$ $CD4^+$ T cells. **B)** Splenocytes were activated with $MOG_{35\text{-}55}$ for 72 h. GM-CSF, IFN-γ, and IL-17A concentrations in cell culture supernatants were measured by ELISA. These experiments were conducted two times with similar outcomes. Data shown are mean ± SEM. P-values were calculated using unpaired Student's *t*-test; * $p < 0.05$, ** $p < 0.01$, NS: not significant.

Supplementary Figure 2

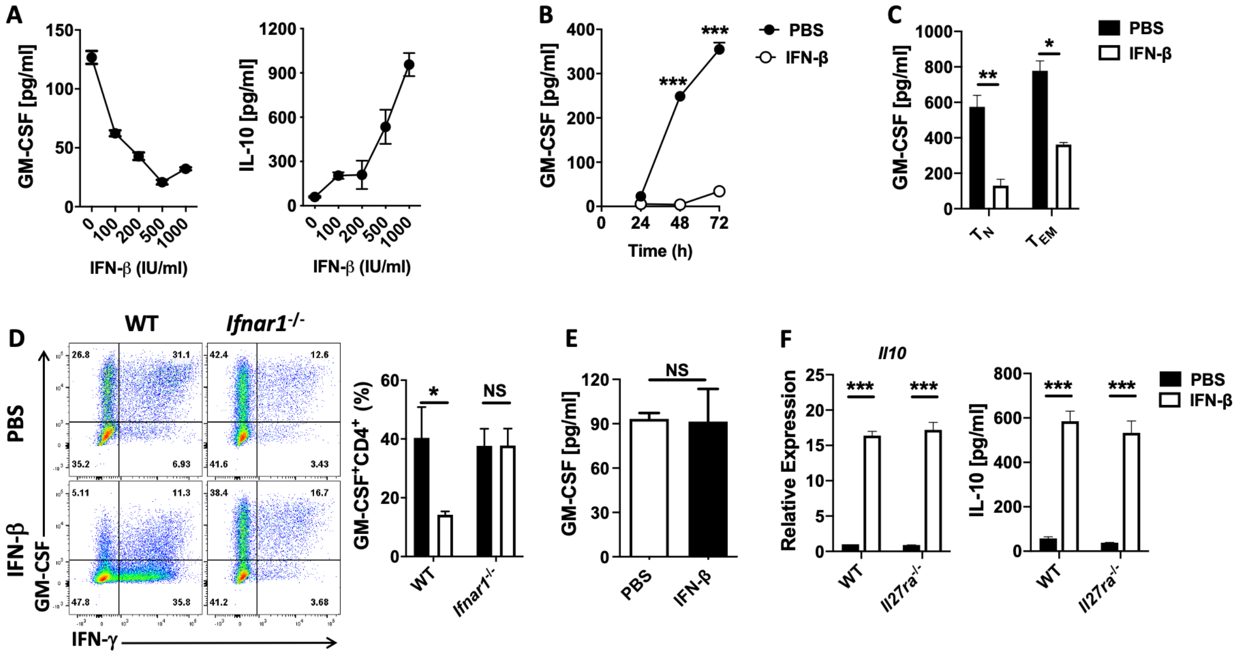

Supplementary Figure 2. rIFN-β acts on antigen presenting cells to suppress GM-CSF production by Th cells. A) Naïve CD4$^+$ T cells were co-cultured with APCs and activated with anti-CD3/28 Abs in different concentrations of rIFN-β for 72 h. GM-CSF and IL-10 concentrations in cell culture supernatants were measured by ELISA. **B)** Naïve CD4$^+$ T cells were co-cultured with APCs, activated with anti-CD3/28 Abs, with or without addition of rIFN-β (1000 IU/ml) in culturing media. GM-CSF concentrations in cell culture supernatants were measured by ELISA at different time points. **C)** Naïve (T_N) or effector/memory (T_{EM}) CD4$^+$ T cells were co-cultured with APCs, activated with anti-CD3/28 Abs, with or without addition of rIFN-β into culturing media. GM-CSF concentrations in cell culture supernatants were measured by ELISA after 72 h of culturing. **D)** WT and *Ifnar1*$^{-/-}$ naïve CD4$^+$ T cells were co-cultured with WT and *Ifnar1*$^{-/-}$ APCs, respectively, and activated for 72 h with anti-CD3/28 Abs, with or without addition of rIFN-β into culturing media. Representative flow cytometry dot plots showing GM-CSF and IFN-γ expression by WT and *Ifnar1*$^{-/-}$ CD4$^+$ T cells, and proportions (%) of GM-CSF$^+$ CD4$^+$ T cells. **E)** CD4$^+$ T cells were isolated from spleen of WT mice and cultured with anti-CD3/28 Abs, with or without addition of rIFN-β into culturing media. GM-CSF concentrations in cell culture supernatants were measured by ELISA after 72 h of culturing. **F)** BM cells from WT and *Il27ra*$^{-/-}$ mice were differentiated into macrophages and either treated or not with rIFN-β for 24 h; RNA was extracted, and IL-10 expression quantified using RT-PCR. IL-10 concentrations in cell culture supernatants were measured by ELISA. These experiments were conducted three times with similar outcomes. Data shown are mean ± SEM.

P-values were calculated using unpaired Student's *t*-test; * $p < 0.05$, ** $p < 0.01$, *** $p < 0.001$, NS: not significant.

Supplementary Figure 3

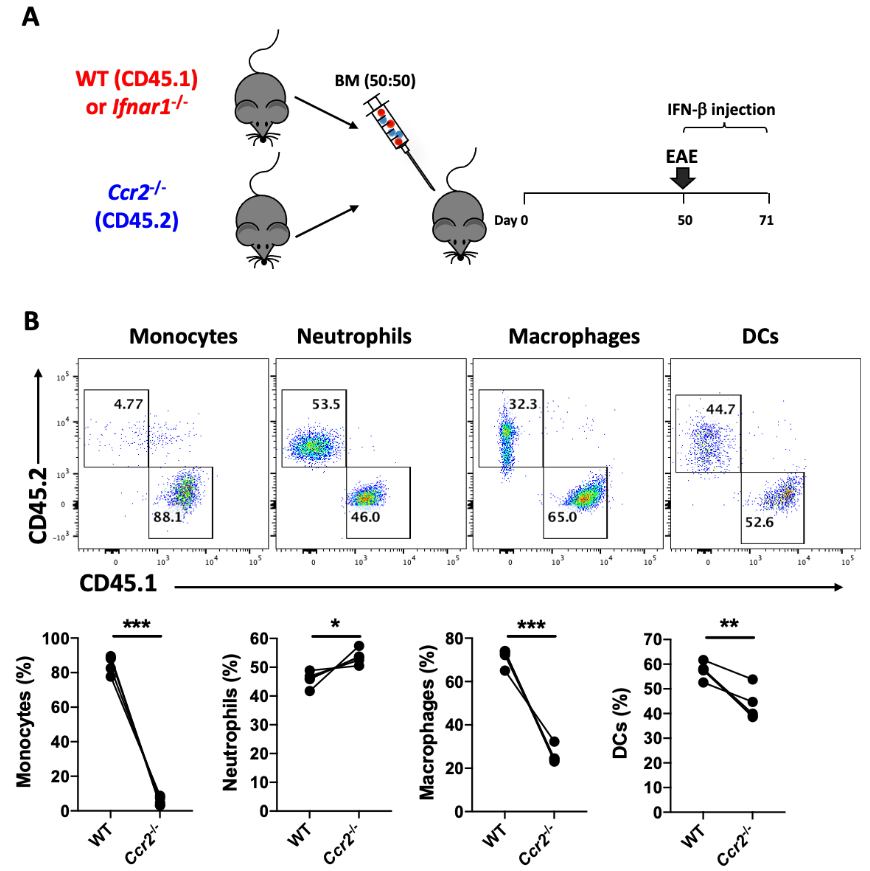

Supplementary Figure 3. *Ccr2*$^{-/-}$ monocytes fail to migrate to the periphery in mixed BM chimera mice. A) Schematic showing generation of mixed BM chimera mice with WT (CD45.1) or *Ifnar1*$^{-/-}$ BM mixed with *Ccr2*$^{-/-}$ (CD45.2) BM in a 1:1 ratio and injected into irradiated WT mice. Recipients were immunized for EAE induction after seven weeks of reconstitution and injected with rIFN-β daily. **B)** After seven weeks of reconstitution, populations of APCs from blood of chimeric mice were analyzed by flow cytometry. Representative flow cytometry dot plots showing distribution of WT (CD45.1) and *Ccr2*$^{-/-}$ (CD45.2) cells among different populations of APCs. Proportions (%) of WT (CD45.1) and *Ccr2*$^{-/-}$ (CD45.2) cells among different populations of APCs. P-values were calculated using paired Student's *t*-test in; *** $p < 0.05$, ** $p < 0.01$, *** $p < 0.001$.

Supplementary Figure 4

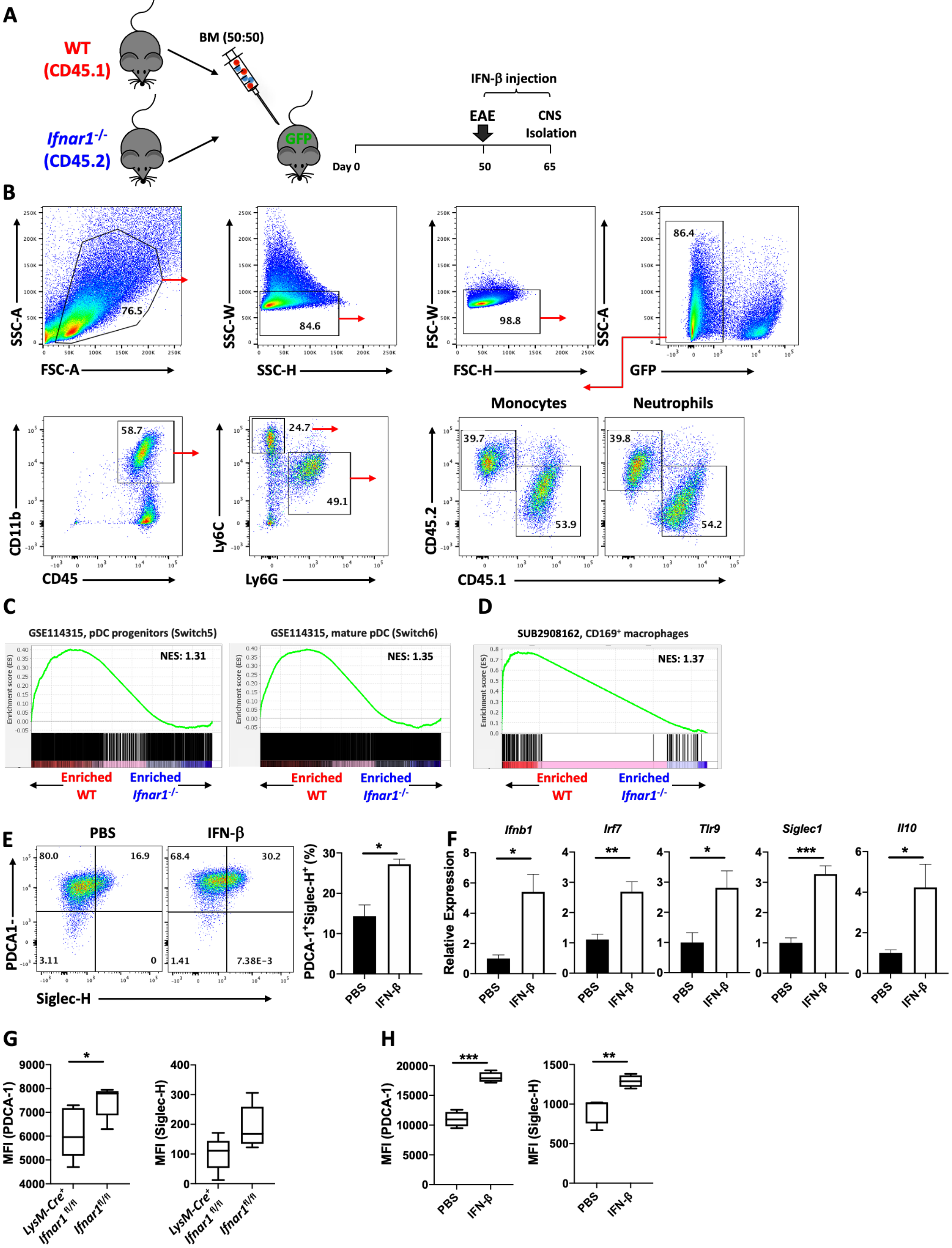

Supplementary Figure 4. rIFN-β increases type-I IFN expressing genes in monocytes from the CNS. A) Schematic showing experimental design with mixed BM chimera mice generated with BM from WT (CD45.1) and *Ifnar1*$^{-/-}$ (CD45.2) mice injected into lethally irradiated GFP^{+} recipient mice. GFP^{+} recipients were immunized for EAE induction after seven weeks of

reconstitution. Mice were sacrificed at disease peak and RNA from CNS monocytes was extracted and analyzed by RNA-seq. **B)** Gating strategies for sorting monocytes (GFP^- $CD45^{hi}CD11b^+Ly6C^{hi}Ly6G^-$) in A). **C)** GSEA enrichment plots of rIFN-β-treated WT monocytes showing upregulated pDC progenitor and mature pDCs (GEO accession no. GSE114315) genes, and **D)** $CD169^+$ macrophage (SRA#SUB2908162) genes. **E)** WT mice were immunized for EAE induction, CNS mononuclear cells isolated at day 15 p.i. and activated for 24 h with MOG_{35-55}, with or without addition of rIFN-β into culturing media. Representative flow cytometry dot plots showing staining of monocytes for PDCA-1 and Siglec-H, and their proportions (%). **F)** Monocytes from E) were FACS sorted and RNA extracted for gene expression analysis by RT-PCR. **G)** CNS monocytes from rIFN-β-treated *Ifnar1*$^{fl/fl}$ and LysM-Cre$^+$*Ifnar1*$^{fl/fl}$ mice with EAE were analyzed by flow cytometry. MFI (mean fluorescent intensity) of staining for PDCA-1 and Siglec-H. **H)** MFI of monocyte staining for PDCA-1 and Siglec-H treated with rIFN-β in E). Box plots showing interquartile range (IQR), with horizontal line denoting median. P-values were calculated using unpaired Student's *t*-test in; *** $p < 0.05$, ** $p < 0.01$, *** $p < 0.001$.

Supplementary Figure 5

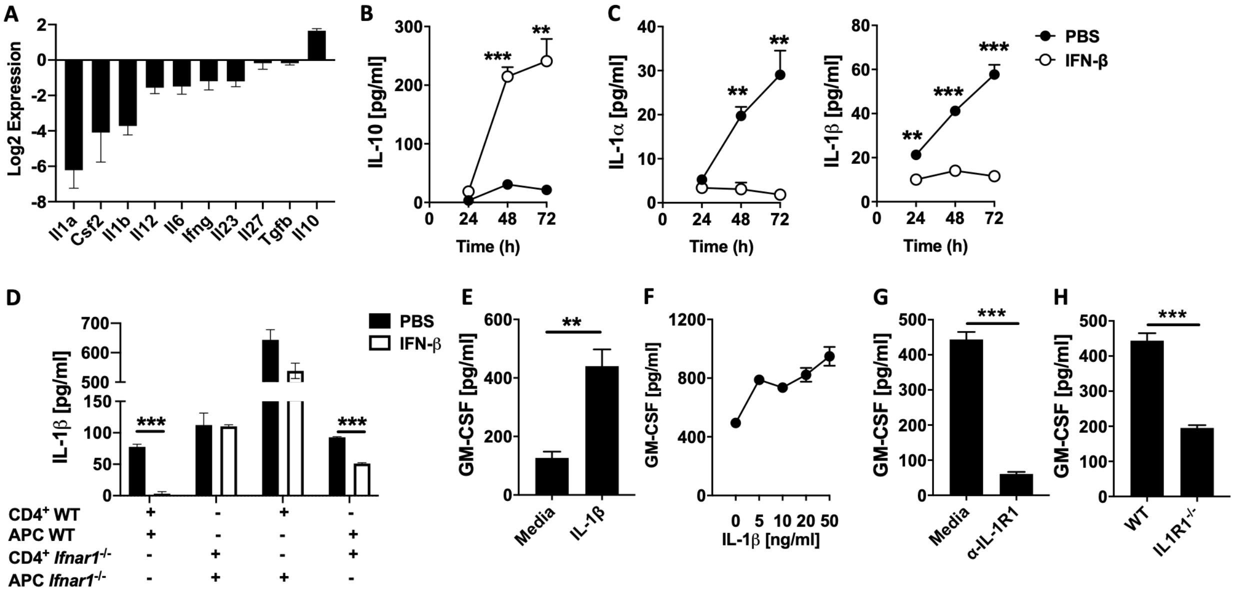

Supplementary Figure 5. rIFN-β suppresses IL-1β production by APCs. Naïve CD4$^+$ T cells were co-cultured with APCs, activated with anti-CD3/28 Abs, with or without addition of rIFN-β in culturing media. **A)** RNA was extracted after 72 h of culturing and gene expression was quantified using RT-PCR. **B)** IL-10 and **C)** IL-1α and IL-1β concentrations in cell culture supernatants were measured by ELISA at different time points of culturing. **D)** WT or *Ifnar1*$^{-/-}$ naïve CD4$^+$ T cells were co-cultured with WT or *Ifnar1*$^{-/-}$ APCs, activated with anti-CD3/28 Abs, with or without addition of rIFN-β into culturing media. IL-1β concentrations in cell culture supernatants were measured by ELISA after three days of culturing. **E-G)** Naïve CD4$^+$ T cells were co-cultured with APCs and activated for 72 h with anti-CD3/28 Abs. GM-CSF concentrations were measured in cell culture supernatants by ELISA. **E)** with or without addition of IL-1β (20 ng/ml); **F)** in several concentrations of IL-1β; **G)** with addition of anti-IL-1R1 Ab in culturing media. **H)** WT and *Il1r1*$^{-/-}$ naïve CD4$^+$ T cells were co-cultured with WT and *Il1r1*$^{-/-}$ APCs and activated with anti-CD3-28 Abs. GM-CSF concentrations in cell culture supernatants were measured by ELISA after 72 h of culturing. Data shown are mean ± SEM. Experiments were conducted at least three time with similar outcomes. For EAE, p-values were calculated using unpaired Student's *t*-test; ** $p < 0.01$, *** $p < 0.001$.

Supplementary Figure 6

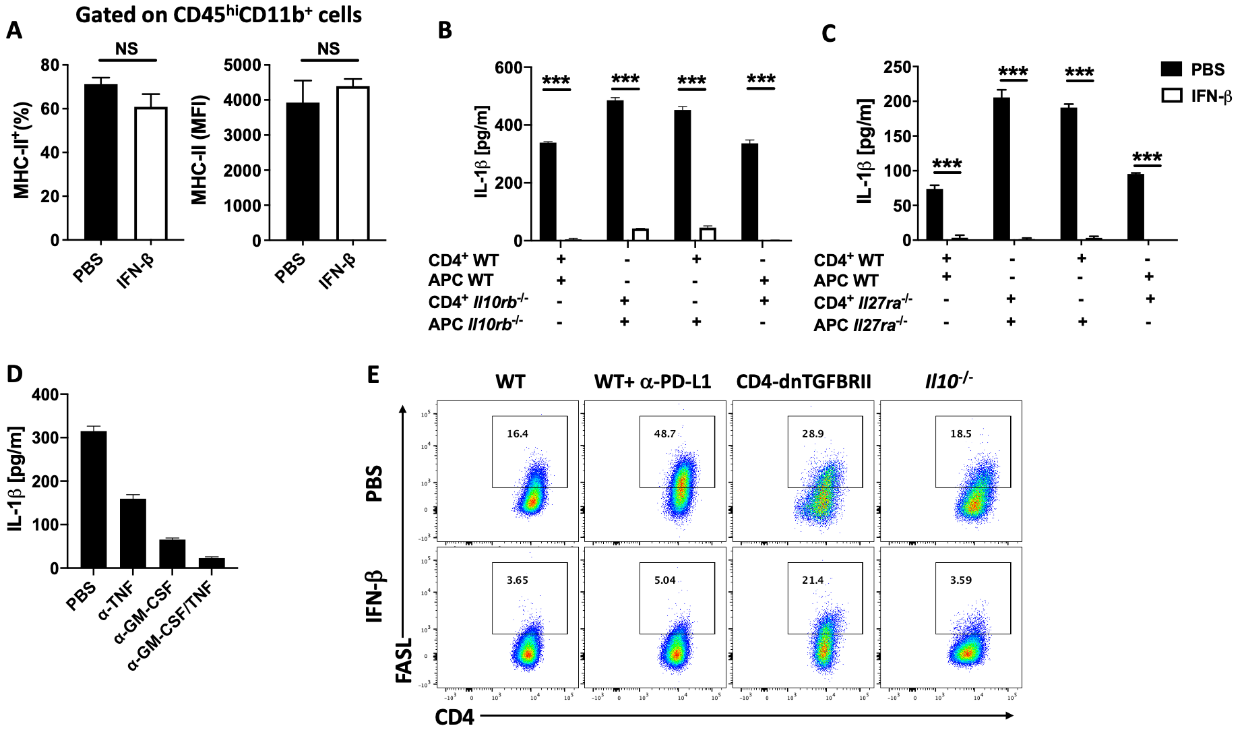

Supplementary Figure 6. rIFN-β suppresses IL-1β production by myeloid cells independently of IL-10 and IL-27 signaling. A) WT mice were immunized for EAE induction and treated daily with PBS or rIFN-β. Mice were sacrificed at day 15 p.i. and CD11b$^+$ cells from the CNS analyzed by flow cytometry for MHC-II expression. Proportions (%) and levels (MFI) of MHC-II expression by myeloid cells are shown. **B)** WT or *Il10rb*$^{-/-}$ naïve CD4$^+$ T cells were co-cultured with WT or *Il10rb*$^{-/-}$ CD11b$^+$ cells, activated with anti-CD3/28 Abs, with or without addition of rIFN-β into culturing media for 72 h. IL-1β concentrations in cell culture supernatants were measured by ELISA. **C)** WT or *Il27ra*$^{-/-}$naïve CD4$^+$ T cells were co-cultured with WT or *Il27ra*$^{-/-}$ CD11b$^+$ cells, activated with anti-CD3/28 Abs, with or without addition of rIFN-β into culturing media. IL-1β concentrations in cell culture supernatants were measured by ELISA. **D)** Naïve CD4$^+$ T cells were co-cultured with CD11b$^+$ cells, and activated with anti-CD3/28 Abs, with or without addition of anti-TNF and anti-GM-CSF Abs into culturing media. IL-1β concentrations in cell culture supernatants were measured by ELISA. **E)** Splenic WT naïve CD4$^+$ T cells were co-cultured with WT CD11b$^+$ cells with or without addition of anti-PD-L1 Ab; CD4-dnTGFBRII and *Il10*$^{-/-}$ naïve CD4$^+$ T cells were co-cultured with WT and *Il10*$^{-/-}$ CD11b$^+$ cells, respectively. Cells were activated with anti-CD3/28 Abs, with or without addition of rIFN-β into culturing media for 72 h. Representative flow cytometry dot plots showing staining of Th cells for FASL are shown. These

experiments were conducted at least two times with similar outcomes. Data shown are mean ± SEM. P-values were calculated using unpaired Student's *t*-test; *** $p < 0.001$, NS: not significant.

Supplementary Figure 7

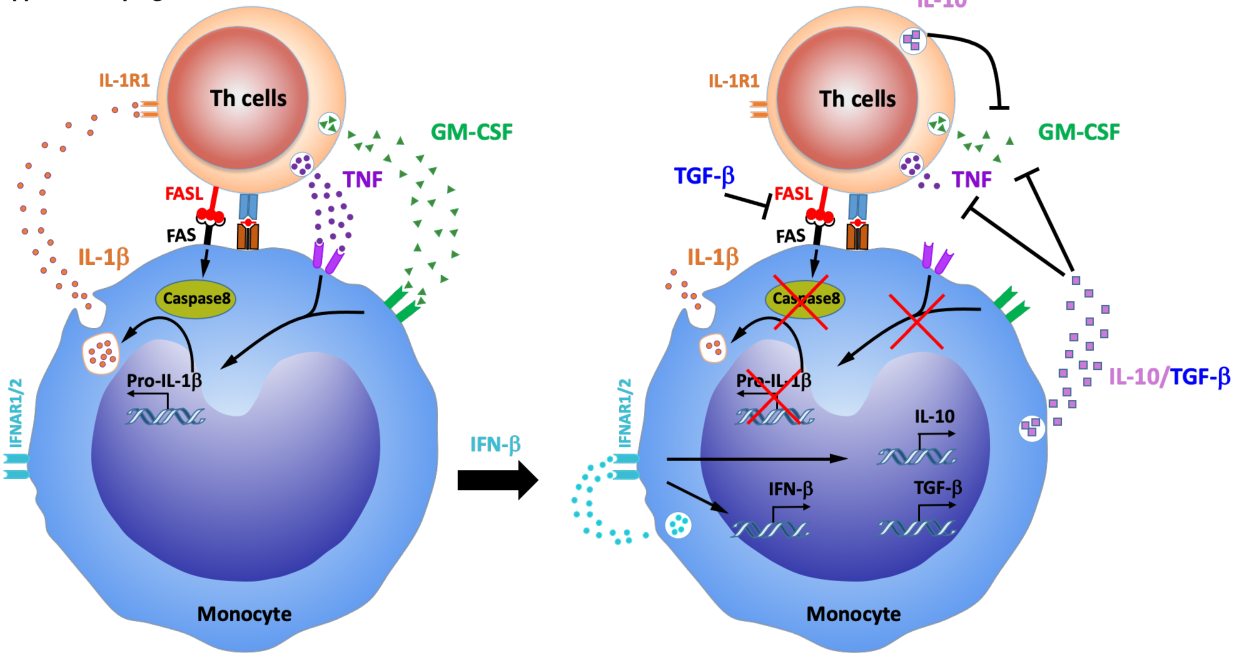

Supplementary Figure 7. rIFN-β acts on monocytes to disrupt the positive feedback loop between monocytes and Th cells. Encephalitogenic Th cells express GM-CSF, TNF, and FASL that induce IL-1β production by monocytes in a caspase 8-dependent manner. rIFN-β suppresses CNS inflammation by acting on monocytes to induce an anti-inflammatory phenotype in them. These monocytes ameliorate CNS inflammation by producing IL-10 and likely TGF-β that suppress the encephalitogenic features of Th cells, namely their GM-CSF, TNF, and FASL expression, resulting in inhibition of a proinflammatory feedback loop between Th cells and APCs.

Table S1. Anti-mouse flow cytometry Abs.

Antigen	Fluorochrome	Clone	Supplier	Category
CD4	Brilliant Violet 785	RM4-5	Biolegend	Surface
CD4	Brilliant Violet 711	RM4-5	Biolegend	Surface
CD4	FITC	RM4-5	BD Biosciences	Surface
CD8a	PerCP/Cy5.5	53-6.7	BD Biosciences	Surface
CD3	DAPI	IM7	Biolegend	Surface
CD45	DAPI	30-F11	Biolegend	Surface
CD45.1	PE-CF594	A20	BD Biosciences	Surface
CD45.2	Brilliant Violet 650	104	Biolegend	Surface
CD19	APC	1D3	BD Biosciences	Surface
CD11b	FITC	M1/70	Biolegend	Surface
CD11c	Brilliant Violet 711	N418	Biolegend	Surface
CD11c	PE/Cy5	N418	Biolegend	Surface
Ly6C	PE	HK1.4	Biolegend	Surface
Ly6C	Brilliant Violet 421	HK1.4	Biolegend	Surface
CCR2	Brilliant Violet 605	SA203G11	Biolegend	Surface
Ly6G	APC-Cy7	1A8	Biolegend	Surface
Ly6G	Brilliant Violet 711	1A8	Biolegend	Surface
MHC-II	FITC	2G9	BD Biosciences	Surface
MHC-II	Brilliant Violet 785	M5/114.15-2	Biolegend	Surface
CD26	FITC	H194-112	Biolegend	Surface
FASL	PE	MFL3	Biolegend	Surface
PDCA-1	Brilliant Violet 650	927	Biolegend	Surface
Siglec-H	PerCP/Cy5.5	551	Biolegend	Surface
GM-CSF	PE	MP1-22E9	Biolegend	Intracellular
GM-CSF	PE-Cy7	MP1-22E9	Biolegend	Intracellular
IL-17A	PE-Cy7	TC11-18H10.1	Biolegend	Intracellular
IL-17A	Brilliant Violet 510	TC11-18H10.1	Biolegend	Intracellular
IFN-γ	APC	XMG1.2	Biolegend	Intracellular
IFN-γ	APC-Cy7	XMG1.2	Biolegend	Intracellular
IL-10	Brilliant Violet 605	JES5-16E3	Biolegend	Intracellular
TNF	FITC	MP6-XT22	BD Biosciences	Intracellular
Pro-IL-1β	APC	NJTEN3	eBiosciences	Intracellular
